# Hydrogel-based nanoparticles: revolutionizing brain tumor treatment and paving the way for future innovations

**DOI:** 10.1186/s40001-025-02310-2

**Published:** 2025-02-04

**Authors:** Alireza Shadab, Simin Farokhi, Arshia Fakouri, Neda Mohagheghzadeh, Ali Noroozi, Zahra Sadat Razavi, Arian Karimi Rouzbahani, Hamidreza Zalpoor, Mohamad Mahjoor

**Affiliations:** 1 Department of Immunology, School of Medicine, Semnan University of Medical Sciences, Semnan, Iran; 2 Deputy of Health, Iran University of Medical Sciences, Tehran, Iran; 3 Student Research Committee, Lorestan University of Medical Sciences, Khorramabad, Iran; 4 USERN Office, Lorestan University of Medical Sciences, Khorramabad, Iran; 5 Department of Bacteriology & Virology, School of Medicine, Shiraz University of Medical Sciences, Shiraz, Iran; 6 Dental Research Center, Faculty of Dentistry, Mazandaran University of Medical Sciences, Sari, Iran; 7 Physiology Research Center, Iran University Medical Sciences, Tehran, Iran; 8 Biochemistry Research Center, Iran University Medical Sciences, Tehran, Iran; 9 Advanced Bioengineering Initiative Center, Computational Medicine Center, K. N. Toosi University of Technology, Tehran, Iran; 10 Shiraz Neuroscience Research Center, Shiraz University of Medical Sciences, Shiraz, Iran; 11 Network of Immunity in Infection, Malignancy & Autoimmunity (NIIMA), Universal Scientific Education & Research Network (USERN), Tehran, Iran; 12 Cellular and Molecular Research Centre, Qom University of Medical Sciences, Qom, Iran; 13 Department of Immunology, Faculty of Medicine, Iran University of Medical Sciences, Tehran, Iran

**Keywords:** Hydrogel, Nanoparticles, Drug delivery, Brain tumor, Glioblastoma, Controlled-release, Blood–brain barrier

## Abstract

Brain tumor treatment remains a significant challenge due to their high mortality and resistance to current therapies. This paper discusses the promising potential of hydrogel-based nanoparticles as innovative drug delivery systems for brain tumor therapy. Extensive characterization techniques reveal the ability of these Nano-systems to demonstrate prolonged blood circulation and targeted delivery, leading to improved survival rates. Designed with optimized physicochemical characteristics, these nanoparticles effectively cross the blood–brain barrier, circumventing a major impediment to drug delivery to the brain. By delivering drugs directly to the tumor bed, these nanoparticles enhance therapeutic outcomes and minimize adverse effects. In addition, this review investigates the techniques for characterizing, visualizing, and modifying these nanoparticles, as well as the standing challenges and promising research avenues for their clinical application. Further investigations are encouraged by this review to investigate potential advancements in hydrogel-based nanoparticle therapeutic approaches for brain tumors. This includes investigating tailored hydrogels, hybrid systems, computational modeling, and the integration of gene therapy and immunotherapy techniques. The study also addresses the need for enhanced synthesis techniques, stability, scalability, and cost-cutting measures to overcome obstacles and advance the clinical use of hydrogel-based nanoparticles in treating brain tumors.

**Graphical abstract:**

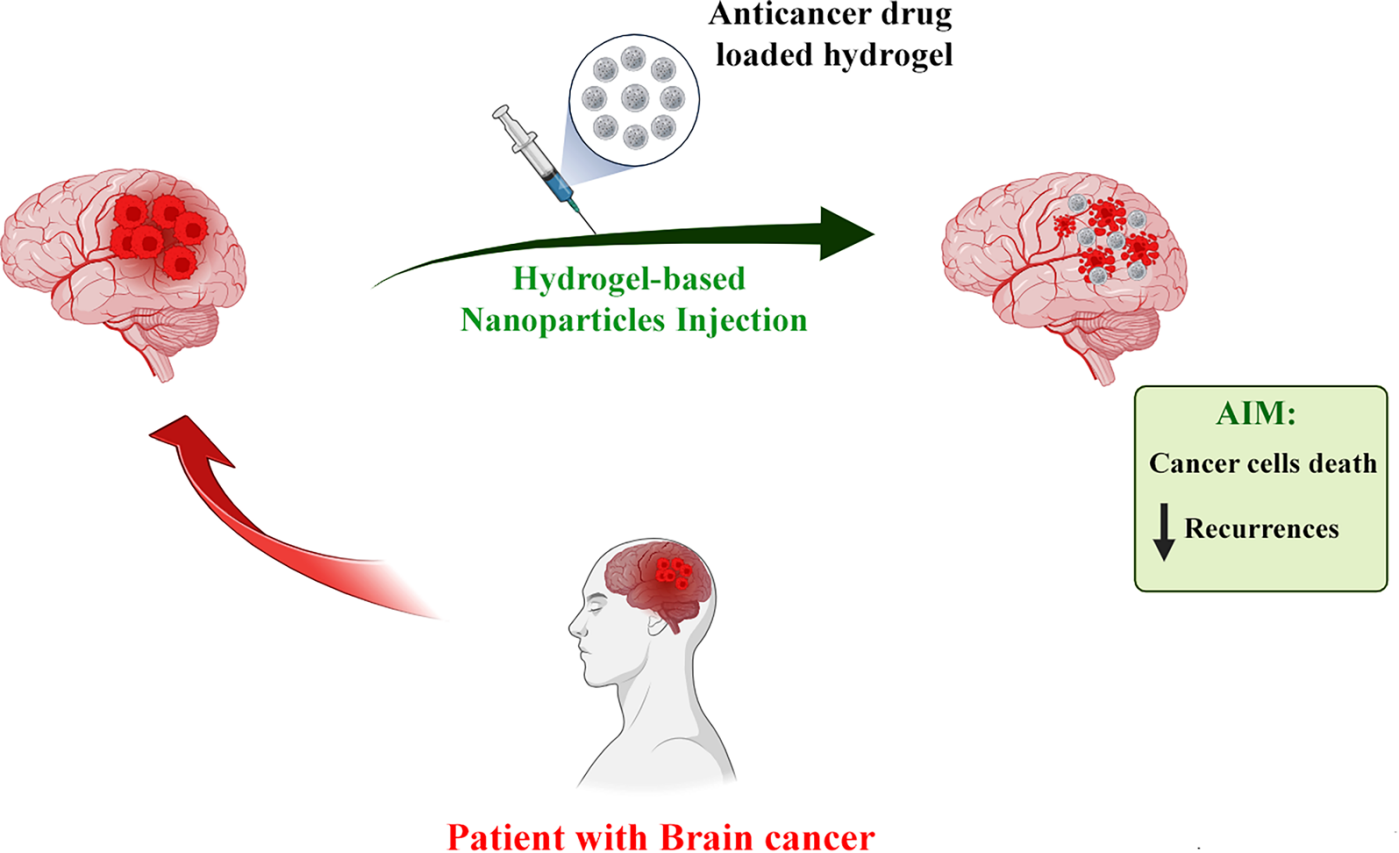

## Introduction

### Overview of brain tumors: prevalence, classification, and clinical challenges

Cancer poses a significant global public health challenge and ranks as the second most common reason for death in the United States. In 2020, disruptions in the diagnosis and treatment of cancer occurred as a result of the effects of the COVID-19 pandemic [1]. Brain tumors (BT) represent a serious and potentially life-threatening condition that garners considerable attention [2]. However, it is crucial to promptly detect and accurately identify the type and location of tumors for treatment effectiveness and the preservation of lives [3]. Tumors of the central nervous system (CNS) stand as the most prevalent solid tumors in childhood, constituting the main reason for cancer-related deaths within this demographic [4]. BT manifests as an abnormal proliferation of cells within or in proximity to the brain, potentially originating in the brain tissue itself or adjacent structures, such as nerves, the pituitary gland, the pineal gland, and the membranes enveloping the brain’s surface. These tumors can be classified as either primary, originating within the brain, or secondary, resulting from the dissemination of cancer cells to the brain from various regions of the body [5]. It can exhibit either a noncancerous (benign) or cancerous (malignant) nature. Noncancerous tumors have the potential to grow gradually, exerting pressure on the brain tissue, whereas malignant tumors may exhibit rapid growth, infiltrating and causing destruction to the brain tissue. The size of brain tumors varies widely, ranging from minute to extensive, with symptoms contingent upon their size and specific location [6]. According to the Central Brain Tumor Registry of the United States, the annual incidence rate of malignant brain tumors has shown a decrease of around 0.8% from 2008 to 2017 across all age groups. Conversely, there has been a notable rise in the occurrence of non-malignant tumors during this period. Between 2014 and 2018, the overall incidence of intracranial neoplasms was recorded at 24.25 cases per 100,000 populations, with malignant brain tumors accounting for 7.06 cases per 100,000 and non-malignant tumors for 17.18 cases per 100,000. This signifies an almost twofold rise in the overall incidence of Brain neoplasms compared to 15 years earlier, when the rate stood at 14.4 cases per 100,000. Projections for the year 2021 estimated that the US would witness the identification of 88,190 new instances of brain and other CNS tumors. This report included 25,690 cases of malignant tumors and 62,500 cases of non-malignant brain tumors. Although constituting less than a third of all BTs account for the majority of deaths related to the disease. The yearly rate of mortality is approximately 4.43 per 100,000, resulting in an average of 16,606 deaths annually from primary malignant brain and other CNS tumors. Gliomas contribute to 78.3% of malignant brain tumors, with glioblastomas (GBM) accounting for more than half of this subgroup. Among non-malignant brain tumors, meningioma is the most prevalent, with pituitary tumors and nerve sheath tumors coming after [7, 8]. Significantly, BTs stand among the prominent contributors to mortality in developed nations. Without appropriate medical intervention, this condition can lead to serious consequences, including visual impairment, speech disorders, and, in some cases, paralysis. Within the realm of medical image analysis, the precise identification and classification of BTs represent pivotal tasks for clinical diagnosis, treatment planning, and disease progression monitoring.

### Benign brain tumors

#### Characteristics

1. **Slow growth:** Benign brain tumors generally exhibit indolent growth kinetics. Tumors, such as meningiomas, which arise from the meningeal layers, can remain asymptomatic for extended periods, with symptoms such as cephalalgia or visual disturbances manifesting only after substantial growth [9].
2. **Well-defined borders:** These tumors typically possess clear demarcation from adjacent parenchyma. This distinct separation facilitates surgical resection. Schwannomas, originating from Schwann cells, and pituitary adenomas, arising from the adenohypophysis, exemplify this characteristic [10].
3. **Non-invasive:** Benign tumors expand without infiltrating surrounding neural structures. This localized growth pattern is exemplified by craniopharyngiomas, which arise near the pituitary gland and exhibit expansive, rather than infiltrative, growth [11].
4. **Low likelihood of spreading:** Benign tumors do not metastasize beyond their site of origin, ensuring a confined therapeutic target. This lack of metastatic potential contrasts sharply with malignant gliomas [12].
5. **Symptoms:** Symptomatology varies based on tumor size and anatomical location. Common presentations include persistent headaches, seizure activity, and focal neurological deficits, such as hemiparesis or sensory loss. The gradual progression of symptoms often mirrors the slow growth of the tumor [13].

#### Treatment methods

1. **Surgical resection:** The primary therapeutic approach for benign tumors involves complete surgical excision. The well-circumscribed nature of these tumors typically allows for gross total resection, significantly improving prognosis. For instance, vestibular schwannomas are often amenable to microsurgical removal [14].
2. **Observation (watchful waiting):** For asymptomatic or minimally symptomatic tumors, particularly those in anatomically sensitive areas, serial imaging and clinical observation may be appropriate. This strategy is frequently employed for small, asymptomatic meningiomas [15].
3. **Radiation therapy:** Stereotactic radiosurgery (SRS), utilizing precise radiation delivery systems, such as Gamma Knife or CyberKnife, is often employed for residual or recurrent tumors. This modality is effective in managing pituitary adenomas and small meningiomas when surgery is contraindicated [16].

### Malignant brain tumors

#### Characteristics

1. **Aggressive nature:** Malignant brain tumors, exemplified by glioblastoma multiforme (GBM), exhibit rapid proliferative rates and extensive neoangiogenesis, contributing to their aggressive clinical behavior and poor prognosis [17].
2. **Invasive:** These tumors demonstrate diffuse infiltration into surrounding brain parenchyma, complicating surgical resection. High-grade gliomas, including anaplastic astrocytomas and oligodendrogliomas, often extend beyond visible margins, necessitating adjuvant therapies [18].
3. **High recurrence rates:** Malignant brain tumors frequently recur post-treatment, driven by residual microscopic disease and inherent resistance mechanisms. GBM, in particular, exhibits nearly universal recurrence despite multimodal therapy [19].
4. **Metastasis:** While primary CNS tumors seldom metastasize extracranially, they can disseminate via cerebrospinal fluid pathways, as seen in medulloblastomas, which can seed the spinal leptomeninges [20].
5. **Symptoms:** Symptomatology is rapid and severe, often including progressive headaches, seizure disorders, cognitive impairment, and profound neurological deficits, such as aphasia or hemiplegia. These manifestations reflect the tumor’s infiltrative and expansive behavior [20].

#### Treatment methods

1. **Surgery:** Maximal safe resection remains the cornerstone of initial management. Intraoperative technologies such as awake craniotomy and fluorescence-guided surgery (e.g., 5-ALA) are employed to enhance resection while preserving neurological function [21].
2. **Chemotherapy:** Alkylating agents like temozolomide are standard in the management of high-grade gliomas, often administered concomitantly with radiotherapy (Stupp protocol) and as maintenance therapy. Ongoing research investigates novel agents and drug delivery systems to overcome the blood–brain barrier [22].
3. **Radiation therapy:** External beam radiotherapy (EBRT) techniques, including intensity-modulated radiation therapy (IMRT) and stereotactic radiosurgery (SRS), are critical for local control. Hypofractionated regimens are explored for their potential to enhance efficacy and reduce treatment duration [23].
4. **Emerging treatments:**
  **Hydrogel-based nanoparticles:** Nanoparticle-encapsulated hydrogels offer targeted, controlled drug delivery, addressing the limitations of conventional chemotherapeutics. These systems can deliver agents like doxorubicin directly to the tumor microenvironment, enhancing local concentration and minimizing systemic toxicity [24]. Preclinical models demonstrate significant tumor penetration and retention, suggesting potential for improved therapeutic outcomes [25]. Hydrogel-based nanoparticles represent an innovative platform for the treatment of brain tumors. Hydrogels, composed of hydrophilic polymer networks, can encapsulate therapeutic agents, allowing for controlled, localized drug delivery [26]. This technology is particularly advantageous in overcoming the blood–brain barrier and achieving high local drug concentrations.
  **Immunotherapy:** Immune checkpoint inhibitors (e.g., nivolumab, pembrolizumab) and CAR-T cell therapies represent promising avenues, leveraging the immune system to target malignant cells. Ongoing trials are assessing their efficacy and safety in gliomas [27].
  **Targeted Therapy:** Agents targeting specific molecular alterations, such as bevacizumab (anti-VEGF) for angiogenesis inhibition and BRAF inhibitors for tumors with specific mutations, are under investigation. Molecular profiling of tumors facilitates personalized treatment strategies [28].

**Hydrogel-based nanoparticles:** Hydrogel-based nanoparticles represent an innovative platform for the treatment of brain tumors. Hydrogels, composed of hydrophilic polymer networks, can encapsulate therapeutic agents, allowing for controlled, localized drug delivery. This technology is particularly advantageous in overcoming the blood–brain barrier and achieving high local drug concentrations [29].

### An innovative approach of hydrogel-based nanoparticles: advantages in brain tumor treatment

The advent of automated segmentation techniques has significantly enhanced the comprehension of medical professionals regarding the volumetric properties and spatial distribution of tumors. This technological development has facilitated the provision of more precise and personalized medical treatments. Nonetheless, it is imperative to underscore the inherent complexities associated with delineating boundaries between normal tissues surrounding pathological tumor areas [30–32]. The approach to treating a malignant brain tumor is contingent upon various factors, including whether the tumor is cancerous or noncancerous, its type, size, grade, and location. The primary treatment modalities encompass:

Treating brain tumors typically includes different approaches, such as surgery, radiation therapy, chemotherapy, targeted drug therapy, and the use of tumor treating fields. In conjunction with these primary treatments, additional interventions may include the prescription of steroids to alleviate swelling around the tumor. Furthermore, other medications may be administered to manage specific symptoms associated with brain tumors, such as anti-epileptic drugs for seizures and pain relievers for headaches [33]. It is crucial to acknowledge that the formulation of a tailored treatment plan for a benign brain tumor hinges on various factors, encompassing the patient’s symptoms, overall health, and treatment preferences. Notwithstanding notable progress in treatment modalities, existing management strategies frequently encounter challenges, particularly in efficiently delivering therapeutic agents through the blood–brain barrier (BBB) and precisely focusing on the tumor without impacting surrounding healthy tissue [34]. Herein lies the potential of an innovative approach: hydrogel-based nanoparticles emerge as a promising frontier for surmounting these challenges. Possessing distinctive physicochemical properties, such as biocompatibility and a high degree of tunability, hydrogel-based nanoparticles can be meticulously engineered to augment drug delivery systems. Moreover, advancements in nanoparticle synthesis provide a robust platform for the development of targeted therapies, holding the potential to markedly enhance treatment outcomes for individuals with brain tumors.

Given these considerations, our focus pivots towards comprehending how hydrogel-based nanoparticles could catalyze a paradigm shift in BT management. This shift signifies a transition from an era characterized by systemic toxicity and limited efficacy to one marked by precision and personalized care. Hydrogel-based nanoparticles represent a subset of nanomaterials that has garnered considerable attention in the realms of nanotechnology and biomedical engineering. These nanoparticles originate from hydrogels, which are polymer networks characterized by water-swelling and high cross-linkages [35]. Hydrogels are soft, quasi-solid materials that have the capability to uphold and safeguard biological substances [35]. Nevertheless, conventional hydrogels exhibit weak mechanical properties and struggle to maintain intricate structures. Addressing these limitations involves the incorporation of nanoparticles into the hydrogel matrix [36]. Nanoparticles play a pivotal role in mechanically reinforcing hydrogels through a combination of physical and chemical interactions. This reinforcement has a positive impact on the 3D printability and structural integrity of the hydrogel by modulating its rheological, biomechanical, and biochemical properties. Consequently, this enhancement provides greater flexibility for printing a diverse array of structures. Furthermore, the introduction of nanoparticles to hydrogels imparts new bio-functionalities, influencing both cell–material and cell–cell interactions within the hydrogel. In addition, nanoparticles contribute to the incorporation of “smart” features, endowing the tissue constructs with responsiveness to external stimuli. This responsiveness encompasses reactions to magnetic fields, electric fields, pH changes, and near-infrared light, facilitated by the integration of nanoparticles. Moreover, hydrogel polymeric networks containing nanoparticles can undergo advanced chemical crosslinking, affording increased flexibility in printing structures with varied biomechanical properties [35]. The versatile applications of hydrogel-based nanoparticles, particularly in the realm of biomedicine, render them highly advantageous. They find applications in diverse areas, such as delivering drugs, tissue engineering, regenerative medicine, and creating innovative bioinks for 3D bioprinting. Specifically in drug delivery, these nanoparticles excel in encapsulating and transporting drugs to precise target sites within the body [37]. In the domains of tissue engineering and regenerative medicine, hydrogel-based nanoparticles are instrumental in fabricating scaffolds that emulate the natural extracellular matrix. These scaffolds play a crucial role in supporting cell growth and facilitating tissue regeneration. In the context of 3D bioprinting, these nanoparticles are employed to formulate bioinks capable of being printed into intricate, cell-laden structures. This not only enhances the overall printability but also introduces novel functionalities, including responsiveness to external stimuli [38]. Furthermore, hydrogel-based nanoparticles find application in 3D bioprinting for the generation of intricate structures laden with cells. This capability facilitates the construction of more realistic models of brain tumors, serving as invaluable tools in the research and development of novel treatments [39]. The potential of hydrogel-based nanoparticles in the treatment of intracranial neoplasm is noteworthy. Their capacity to encapsulate and deliver drugs to precise target sites within the body holds the promise of enhancing the efficacy of chemotherapy. This improvement can result in better treatment outcomes while concurrently minimizing side effects. The targeted delivery mechanism enables a heightened concentration of the drug to be directly administered to the tumor cells [40, 41]. Furthermore, the utilization of hydrogel-based nanoparticles extends to the creation of scaffolds that mimic the natural extracellular matrix, fostering a conducive environment for cell growth and tissue regeneration. This application holds particular promise in the context of brain tumor treatment, where it can contribute to the replacement of damaged or compromised brain tissue [42, 43]. Treating brain tumors poses significant challenges, primarily attributable to their heightened invasiveness, heterogeneity, and resistance to conventional therapeutic approaches. Compounding these challenges is the formidable presence of the blood–brain barrier, which restricts the delivery of most drugs to the brain. This limitation diminishes the therapeutic efficacy of drugs and concurrently elevates systemic toxicity [44, 45]. Hence, there arises a necessity for innovative strategies to surmount these challenges and enhance the prospects for individuals with brain tumors. A particularly promising avenue involves the adoption of hydrogel-based drug delivery nanosystems. These systems amalgamate the benefits of hydrogels and nanoparticles, aiming to realize localized, controlled, and targeted delivery of anticancer agents specifically to BTs [46]. Hydrogels represent three-dimensional polymeric networks capable of absorbing substantial quantities of water, mirroring the structure of the extracellular matrix found in tissues. These hydrogels can be engineered to exhibit responsiveness to diverse stimuli, including changes in temperature, pH, light exposure, or magnetic fields. This unique characteristic enables the controlled release of drugs in a spatiotemporal manner [47, 48]. Nanoparticles, as carriers at the nanoscale, offer the potential to improve the solubility, stability, and specificity of drugs. They possess the capability to traverse the BBB through various mechanisms, including receptor-mediated transcytosis, adsorptive-mediated transcytosis, and the enhanced permeability and retention effect. This allows for enhanced drug delivery to the brain with increased precision [49]. The integration of nanoparticles into hydrogels facilitates the development of multifunctional nanosystems. These systems exhibit the capability to deliver drugs to brain tumors with increased efficiency and selectivity, thereby overcoming the constraints associated with conventional chemotherapy [50, 51].

Numerous studies have showcased the potential of hydrogel-based drug delivery nanosystems in the treatment of brain tumors, with evidence from both in vitro and in vivo [52]. As an illustration, there have been endeavors to synthesize and characterize a self-healing hydrogel designed for injection into the brain. This hydrogel exhibits the ability to release drugs in response to external stimuli, such as light or magnetic fields. Loaded with nanoparticles containing doxorubicin—an extensively employed chemotherapeutic agent—and iron oxide—a magnetic and photothermal agent—this innovative approach showcases the potential for advanced drug delivery systems [53, 54]. The study revealed that using the hydrogel led to a decrease in tumor size and prolonged the survival period in rats with glioblastoma multiforme (GBM), the most common and aggressive type of brain tumor [55]. An additional illustration involves a study that formulated an innovative hydrogel-based nanosystem utilizing polyethylene glycol (PEG) and chitosan (CS) for delivering temozolomide (TMZ), the standard of care for glioblastoma multiforme (GBM). The nanosystem demonstrated heightened drug loading, controlled release, and enhanced antitumor efficacy in both in vitro and in vivo settings when compared to free TMZ. Furthermore, the study demonstrated that the nanosystem’s ability to traverse the BBB and accumulate in tumor tissue, as verified through fluorescence imaging [56, 57]. A third notable example involves the fabrication of a hydrogel-based nanosystem incorporating paclitaxel (PTX), a potent anticancer drug, and gold nanoparticles (AuNPs) serving as photothermal agents to augment drug release upon near-infrared irradiation. This nanosystem exhibited noteworthy characteristics, including high drug loading, sustained release, and superior cytotoxicity against glioblastoma multiforme (GBM) cells in vitro [58]. Furthermore, the nanosystem demonstrated exceptional biocompatibility and favorable biodistribution in vivo. Notably, it significantly impeded tumor growth and prolonged the survival of mice afflicted with orthotopic glioblastoma multiforme (GBM) [59]. These studies exemplify the viability and efficacy of hydrogel-based drug delivery nanosystems for treating brain tumors, suggesting their potential as a valuable alternative to conventional chemotherapy. Nonetheless, further research is imperative to optimize their design, characterization, and evaluation, addressing potential challenges and limitations in their clinical translation. In summary, hydrogel-based nanoparticles present a versatile and promising approach for managing brain tumors. Their capacity to deliver drugs in a targeted manner, support tissue regeneration, and generate realistic 3D tumor models positions them as valuable tools in the ongoing battle against brain cancer. Hydrogel-based nanoparticles have proven successful in various fields, especially in the fields of medicine and drug delivery. This is due to their ability to biodegrade, be compatible with biological systems, have adjustable mechanical strength, bind molecular structures, and respond uniquely to specific stimuli, such as ionic concentration, pH, and temperature [60]. In the realm of BT treatment, hydrogel nanoparticles exhibit promising potential. One notable example involves the design of a hydrogel loaded with nanoparticles, specifically crafted to infiltrate and reprogram macrophages [61]. An additional illustration involves the utilization of a nanoparticle treatment administered intrathecally, facilitating direct delivery between the leptomeninges that safeguard the cerebrospinal fluid [62]. When contrasting traditional treatment approaches with those employing hydrogel nanoparticles, the latter presents several notable advantages. Hydrogel nanoparticles offer a distinctive solution to challenges associated with conventional methods, primarily for three key reasons:

Nanoparticles are instrumental in enhancing the mechanical properties of hydrogels, contributing to their reinforcement through a combination of physical and chemical interactions. In addition, the incorporation of hydrogel nanoparticles facilitates the introduction of novel bio-functionalities, thereby augmenting the overall capabilities of the hydrogels. Furthermore, the integration of nanoparticles into hydrogel-based bioinks imparts “smart” features to these materials, endowing tissue constructs with the ability to respond to external stimuli. This responsiveness adds a layer of precision and adaptability to the treatment process, underscoring the potential significance of nanoparticle incorporation in advancing hydrogel applications in various biomedical contexts [35].

In an overarching assessment, hydrogel-based drug delivery methods demonstrate superior efficacy in cancer treatment compared to conventional systemic chemotherapy. The unique properties of hydrogel-based approaches, such as targeted and controlled drug delivery, enhanced tissue specificity, and the ability to overcome barriers, such as the blood–brain barrier, contribute to their heightened effectiveness in managing cancer [63]. Indeed, one of the notable advantages of hydrogel-based drug delivery methods is their ability to mitigate side effects. By providing a controlled and targeted release of chemotherapeutic drugs directly to the tumor site, these methods minimize systemic exposure and reduce the occurrence of adverse effects associated with conventional systemic chemotherapy. This targeted approach enhances the therapeutic impact on cancer cells while sparing healthy tissues from unnecessary exposure to cytotoxic agents [64]. The focus on controlled-release biodegradable hydrogels as drug delivery approaches for chemotherapy highlights their importance. While these hydrogels offer considerable promise, further research is essential to gain a comprehensive understanding and optimize their use across various applications. In the realm of brain tumor management, hydrogel-based nanoparticles have emerged as a particularly promising tool, thanks to their unique properties and potential applications. Continued exploration of these innovative approaches has the potential to significantly advance strategies for cancer treatment.

Targeted drug delivery, as facilitated by encapsulation within hydrogel-based nanoparticles, represents a significant advancement in chemotherapy. This approach enables the specific and precise delivery of drugs to predetermined target sites within the body. The targeted delivery mechanism significantly improves the efficacy of chemotherapy, resulting in enhanced treatment outcomes while concurrently reducing side effects. This strategy allows for a heightened concentration of the drug to be directly administered to the tumor cells, maximizing its impact on cancerous tissues [65]. In the domain of tissue engineering and regenerative medicine, hydrogel-based nanoparticles play a crucial role in fabricating scaffolds that emulate the natural extracellular matrix. These scaffolds provide a supportive environment conducive to cell growth and tissue regeneration. This application holds particular promise in the context of BT treatment, where it can contribute to the replacement of damaged or compromised brain tissue. The use of hydrogel-based nanoparticles showcases their potential in promoting regenerative processes and addressing tissue damage associated with conditions, such as brain tumors [66]. In the realm of 3D bioprinting, hydrogel-based nanoparticles play a pivotal role in constructing intricate, cell-laden structures. This capability facilitates the development of more realistic models of brain tumors. These models, created through 3D bioprinting with hydrogel-based nanoparticles, become valuable tools in the research and development of new treatments for brain tumors. By replicating the complex architecture and cellular composition of actual tumors, these models contribute to a better understanding of the disease and aid in the exploration of innovative therapeutic approaches [35]. The inherent biocompatibility of hydrogels is a distinctive feature that renders them suitable for use within the body. Hydrogels can be specifically designed to exhibit compatibility with biological systems, minimizing the risk of adverse reactions. In addition, their capacity to degrade over time is a valuable characteristic, as it reduces the likelihood of long-term complications. This biocompatibility, coupled with controlled degradation, enhances the safety profile of hydrogels, making them favorable candidates for various biomedical applications, including drug delivery and tissue engineering [67]. The ability to achieve controlled drug release is a notable attribute of hydrogel-based nanoparticles. Through precise design, these nanoparticles can release drugs in a controlled manner over a specific period. This controlled drug release offers several advantages, including enhanced treatment effectiveness and a reduction in the frequency of drug administration. By tailoring the release kinetics of drugs, hydrogel-based nanoparticles contribute to optimizing therapeutic outcomes while potentially improving patient compliance and minimizing side effects [68].

## Synthesis and characterization of hydrogel-based nanoparticles

Hydrogels have emerged as a promising platform for drug delivery systems, particularly in the treatment of brain tumors [41]. The selection of appropriate biomaterials for hydrogel preparation is critical, as these materials significantly influence the hydrogels’ properties, including biocompatibility, mechanical strength, and drug release profiles [69, 70].

Natural Polymers are frequently utilized in hydrogel formulations due to their inherent biocompatibility and biodegradability [71]. Alginate, derived from brown seaweed, is widely recognized for its ability to form hydrogels through ionic cross-linking, allowing for the encapsulation of various therapeutic agents while maintaining their stability during delivery [72]. Chitosan, obtained from chitin in crustacean shells, exhibits excellent biocompatibility and can be tailored for drug release by modifying its degree of deacetylation and molecular weight, making it suitable for targeted delivery applications [73]. Chitosan hydrogels can be produced through different methods, such as physical interactions or chemical cross-linking, resulting in a variety of geometries, formulations, and shapes [73]. Another natural polymer, gelatin, forms hydrogels through thermal or chemical cross-linking and promotes cell adhesion and proliferation, which is beneficial for both drug delivery and tissue engineering applications [74].

Synthetic Polymers also play a vital role in hydrogel preparation. Polyethylene glycol (PEG) is a synthetic polymer known for its hydrophilicity and low toxicity. PEG-based hydrogels can be engineered to have precise mechanical properties and degradation rates, allowing for customized drug release profiles that enhance therapeutic efficacy [75]. Similarly, polyvinyl alcohol (PVA) forms hydrogels through physical or chemical cross-linking and is characterized by good mechanical strength and water retention properties, making it suitable for sustained drug delivery systems [76].

Recent advancements have led to the development of hybrid hydrogel systems that combine natural and synthetic polymers [77, 78]. These hybrid systems leverage the advantages of each component to enhance mechanical properties while maintaining favorable biological interactions [78]. For instance, incorporating nanoparticles into hydrogel matrices can improve structural integrity and facilitate controlled drug release [79]. This innovative approach not only addresses the limitations of conventional hydrogels but also enhances their application in overcoming the blood–brain barrier (BBB) and achieving high local drug concentrations at tumor sites [41].

Indeed, hydrogel-based nanoparticles represent a forefront in biomaterials research, ushering in a new era for drug delivery systems, tissue engineering, and biosensing. These innovative nanoparticles captivate attention not only for their diverse potential applications but also for their distinctive capability to replicate the natural extracellular matrix. This remarkable attribute creates a nurturing environment that not only supports cell growth but also facilitates the controlled release of therapeutic agents. The multifaceted nature of hydrogel-based nanoparticles positions them as key contributors to advancing various fields within biomedicine and beyond [80]. The synthesis of hydrogel-based nanoparticles stands as a testament to the sophistication of modern science, employing advanced polymerization techniques to cross-link monomers into a resilient three-dimensional network. Noteworthy is the synthesis of nanoparticles derived from poly (N-isopropyl acrylamide-co-methacrylic acid) through free radical polymerization. This process offers a high degree of customization, allowing for the fine-tuning of properties by adjusting the molar ratios of the monomers. This adaptability ensures that hydrogel-based nanoparticles can be precisely tailored to address the specific requirements of diverse biomedical applications, establishing them as a remarkably versatile tool in the ongoing pursuit of advancing human health [81]. Researchers employ a diverse array of synthesis methodologies, each distinguished by its unique benefits and potential applications. This compendium offers a concise exploration of the various approaches to nanoparticle synthesis, shedding light on the multifaceted nature of this research domain. The choice of a specific synthesis technique is contingent upon the intended application and the inherent properties desired in the nanoparticles. This underscores the customized approach adopted in contemporary nanomaterial fabrication, where the selection of synthesis methods is intricately linked to the targeted functionalities and applications of the resulting nanoparticles. Physical methods for nanoparticle synthesis encompass a variety of techniques, such as high-energy ball milling, laser ablation, electro-spraying, and physical vapor deposition. These methodologies typically entail the physical manipulation of materials to generate nanoparticles without altering their chemical compositions. The use of physical methods provides researchers with precise control over the size, structure, and properties of the resulting nanoparticles, making these techniques valuable for tailored nanomaterial fabrication [82]. Chemical methods constitute one of the most diverse and extensively utilized approaches for nanoparticle synthesis. Among these methods, the sol–gel process holds prominence, involving hydrolysis and polycondensation reactions of molecular precursors to generate a colloidal suspension (sol), which subsequently undergoes gelation to form nanoparticles. Other chemical methods include chemical vapor deposition, metal–organic decomposition, and wet synthesis. The versatility of chemical methods allows for a wide range of nanoparticle compositions and structures, making them a fundamental choice in the synthesis of nanomaterials for various applications [83, 84]. Biological methods entail the utilization of biological systems or their components, including enzymes, proteins, or microorganisms, to synthesize nanoparticles. These approaches are often regarded as more environmentally friendly and can yield nanoparticles with unique properties that are challenging to achieve through physical or chemical means. Biological synthesis methods leverage the inherent processes of living organisms, allowing for the creation of nanoparticles in a manner that aligns with sustainable and eco-friendly practices. The versatility of biological methods opens up new avenues for the development of nanomaterials with distinctive characteristics for various applications [85].

Each of these methods can be tailored to produce hydrogel-based nanoparticles with specific characteristics, including size, shape, surface charge, and functionalization, catering to diverse applications in medicine, electronics, and environmental science. The selection of a synthesis method hinges on the desired properties of the final product and the intended application for the nanoparticles.

To ensure the suitability of hydrogel-based nanoparticles for specific applications, rigorous characterization techniques are essential. Here’s an overview of key techniques employed for the evaluation of these nanoparticles: In the study of nanoparticles, several techniques are employed to understand their physical attributes. Dynamic Light Scattering (DLS) is used to measure the size distribution of nanoparticles in a suspension by analyzing the scattering of light caused by particles in motion. This method provides valuable insights into the size and shape of the particles. For a more detailed analysis at the nanoscale, Transmission Electron Microscopy (TEM) is utilized. This technique offers high-resolution images that enable the determination of the size and shape of nanoparticles. It provides detailed insights into the morphology and structural characteristics of hydrogel-based nanoparticles, contributing to a comprehensive understanding of their physical attributes. In addition, the surface charge of nanoparticles, a critical factor for predicting their stability in suspension and their interaction with biological systems, is assessed through Zeta Potential Analysis. This analysis measures the electric potential at the nanoparticle’s surface, providing valuable information about the colloidal stability and behavior of hydrogel-based nanoparticles, particularly in biological and environmental contexts [86–88]. The chemical composition of nanoparticles is determined using a variety of techniques. Fourier Transform Infrared Spectroscopy (FTIR) is one such method that identifies functional groups and chemical bonds within the nanoparticles, thereby confirming their composition. This technique is particularly useful for understanding the chemical structure and interactions present in hydrogel-based nanoparticles [81]. In addition to FTIR, Nuclear Magnetic Resonance (NMR) is also employed. NMR provides detailed information about the molecular structure and composition of the nanoparticles. It is a powerful tool for elucidating the chemical makeup of hydrogel-based nanoparticles, offering insights into their internal structure and molecular arrangements [81].

**Fig. 1:**
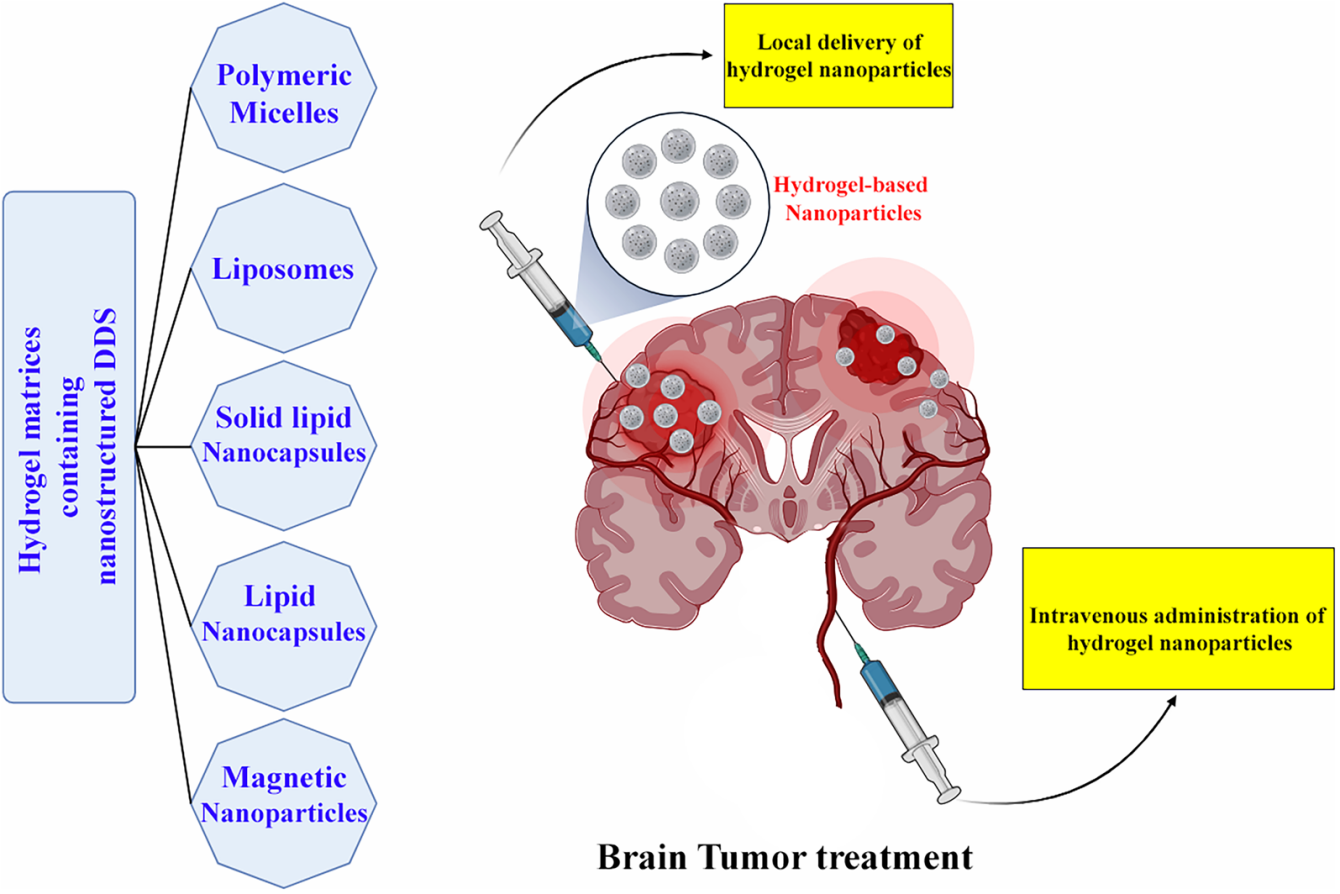
Schematic representation of hydrogel-based nanoparticles for brain tumor treatment. Local delivery of hydrogel nanoparticles loaded with various drug delivery systems (DDS), such as polymeric micelles, liposomes, solid lipid nanocapsules, lipid nanocapsules, and magnetic nanoparticles, via intratumoral injection or implant after surgical resection. This method allows for a controlled and localized release of the therapeutic agent over time. Intravenous administration of hydrogel nanoparticles with surface functionalization as an active targeting strategy. The functionalized nanoparticles can cross the blood–brain barrier and selectively bind to the tumor cells, enhancing the therapeutic efficacy and reducing the side effects

Mechanical Properties, such as: Rheometry: Evaluates the viscoelastic properties of hydrogel nanoparticles, offering insights into their mechanical behavior under stress. This technique is crucial for understanding how hydrogel-based nanoparticles respond to various forces and deformations, providing valuable information about their mechanical stability and suitability for specific applications, particularly in the context of drug delivery and tissue engineering [89]. Indeed, the combination of these characterization techniques forms a comprehensive toolkit for profiling hydrogel-based nanoparticles. This allows researchers to gain a detailed understanding of their size, shape, surface charge, chemical composition, and mechanical properties. By leveraging this wealth of information, researchers can refine and tailor the synthesis of hydrogel-based nanoparticles to meet the specific requirements of diverse applications, ranging from drug delivery to tissue engineering and beyond. The comparison of various hydrogel systems for brain tumor management represents a crucial focus in research, given their distinct advantages in the treatment and diagnosis of brain cancers. Hydrogels offer biomimetic support suitable for both in vitro modeling of brain tumors and therapeutic applications. One notable category is: Engineered Hydrogels: Engineered hydrogels are specifically designed to emulate the microenvironment of brain tumors. This design enables the controlled culture of brain tumor cells, providing a platform for more accurate in vitro modeling. Integration with microscale technologies such as electrospinning and bioprinting enhances the capability to create precise and realistic models of tumor tissues. This approach holds promise for advancing our understanding of brain tumors and developing targeted therapeutic strategies [90]. Drug Delivery Systems: Hydrogel-based drug delivery systems (DDS) are engineered to address the limitations associated with traditional chemotherapy, particularly off-target toxicity. These systems are designed to encapsulate drugs, allowing for localized impact, reduced side effects, and controlled, prolonged drug release. An illustrative example of clinically approved hydrogel systems for brain cancer treatment is Carmustine-loaded copolymers, commonly referred to as “Gliadel wafers.” These hydrogel-based systems have demonstrated efficacy in providing targeted drug delivery within the brain, showcasing the potential of hydrogel technology in enhancing the precision and effectiveness of brain cancer treatment [91] (Fig. 1). Nanogels, also known as hydrogel nanoparticles, present a versatile platform for systemic administration with inherent loading and targeting properties. These nanogels hold the potential to selectively identify and effectively eliminate tumor cells, indicating a hopeful approach for both the treatment and diagnosis of brain cancer. The combination of their size, structure, and drug-loading capabilities positions nanogels as a valuable tool in advancing targeted therapies for brain tumors [41]. Hybrid Hydrogels: Hybrid hydrogels leverage a combination of different materials to enhance their functionality. In the context of brain tumor management, these hybrid systems can be customized to align with the mechanical properties of the brain. By doing so, they create a more physiologically relevant microenvironment for cells, offering a platform that closely mimics the natural conditions within the brain. This tailored approach enhances the potential of hybrid hydrogels for applications related to brain tumor research, diagnostics, and therapeutic interventions [92]. Absolutely, the selection of a hydrogel system for brain tumor management is contingent on several factors, including the type of brain tumor, the intended therapeutic goals, and the individualized needs of the patient. Ongoing research endeavors focus on exploring and comparing different hydrogel systems to optimize strategies for the management of brain tumors. This iterative process of investigation and comparison contributes to the continual refinement and development of innovative approaches in the field of brain cancer treatment.

## Targeting of hydrogel-based nanoparticles for brain tumors management

Surface modifications for targeting specific cell types, such as tumor-associated antigens and receptors.

Enhancing the biological performance of hydrogel nanoparticles is achievable through surface modification. This is demonstrated by a prior study, where Alg was utilized to coat the surface of CS-based hydrogel nanoparticles [93]. In contrast to the uncoated nanoparticles, which released 40% of the loaded enoxaparin within the initial 2 h of treatment in an acidic environment, the coated nanoparticles released only about 2% of the loaded drug [93]. Moreover, the coated nanoparticles can be more efficiently absorbed by the intestine, leading to significantly enhanced anticoagulant activity when orally administered to a rat model with venous thrombosis compared to the activity achieved by the uncoated counterparts [93]. This emphasizes how crucial surface characteristics are to a nanoparticulate system’s functionality. Integrating PEG or other hydrophilic polymers into the surface of the nanoparticle is one method for modifying its surface characteristics [94]. This can delay the mononuclear phagocyte system’s clearance of the hydrogel nanoparticles by reducing non-specific interactions between the particles and serum proteins. The surface of the nanoparticles can also be bound by other ligands, such as transferrin [95], galactose [96], and folic acid [97, 98], to increase the accumulation of the nanoparticles at the target site. Either ligand binding to the polymer components of the nanoparticles or microfabrication technologies can be used to achieve this. To illustrate the latter, a microfluidic device with a component dubbed a “particle exchanger” is used to regularly alter the surface of micro- and nanoparticle in flows [99]. In the exchanger, dielectric particles are subjected to a dielectrophoretic force perpendicular to the fluid flow, where two fluidic channels intersect in a brief area. In this configuration, one cycle of surface modification is completed by the particles returning to a clean buffer after first moving from a buffer solution to a reagent. This technique could make hydrogel nanoparticle surface engineering easier for in vivo and therapeutic uses [100].

In vivo targeting and localization of hydrogel-based nanoparticles, and the impact of these modifications on the pharmacokinetics and biodistribution of the nanoparticles.

Hydrogel nanoparticles, also known as nanogels, are three-dimensional structures composed of crosslinked hydrophilic polymer chains that hydrate in water-based solutions. While their properties resemble those of biological tissue, nanogels are administered systemically and possess specific loading and targeting characteristics within their structure [41]. Microspheres are spherical nanoparticles ranging in size from 1 to 1000 µm. They are employed to encapsulate small drugs and proteins, consequently enhancing the bioavailability, stability, and specificity of these agents [101].

A novel nanogel with surface modification and loaded with cisplatin (CIS) was created to address the overexpression of membrane proteins connexin 43 (Cx43) and brain-specific anion transporter 1 (BSAT1) in glioma and peritumoral cells. PEG-b-poly(methacrylic acid) and MAL–PEG–NH2 were used as starting ingredients to generate hydrogel nanoparticles, which were then conjugated with antibodies and CIS [102]. After the nanogels were unloaded, their diameter measured 120–130 nm, their polydispersity index was 0.13, and their zeta potential was – 15 ± 5 mV [102]. These values indicated that the nanogels could be loaded with a CIS with a capacity of 30–35% and an entrapment efficiency of 45%. In addition, these particles showed persistent release; after a week, around half of the CIS was released. Research on cells revealed that these nanogels were less harmful to C6 cells than free CIS [102]. Nevertheless, the overall survival of rats treated with these nanoparticles and implanted with glioma 101/8 cells was increased, according to in vivo studies. Antibody–receptor interactions are expected to have two functions: they are expected to target the hydrogel nanoparticles and promote the persistence of high levels of CIS close to the tumor [102].

## Drug delivery with hydrogel-based nanoparticles

### Loading and release of drugs from hydrogel-based nanoparticles, with a focus on brain-penetrating drugs

In previous decades, cancer therapy heavily depended on drugs derived from various bacteria and viruses, sourced from biological origins. However, these bio-based drugs, among others, faced challenges as they were prone to easy degradation, rendering them inactive upon administration into the body without effectively reaching the affected region [103]. Therefore, the imperative for successful drug delivery to target sites and the controlled release of drugs is crucial. Hydrogels offer a means to achieve controlled and sustained drug release at the target site by designing them to swell and de-swell in response to specific stimuli or slight changes in conditions, such as temperature or pH alterations [104].

Diffusional mass transport is thought to be the most important mechanism in this context and is principally responsible for the process of physically regulated drug release [105–107] Considering the mass balance, along with initial and boundary conditions, and incorporating certain basic assumptions (such as the negligible erosion of the delivery system during drug release and the constant diffusivity of the diffusing species) [108], models have been developed to describe the kinetics of drug release from diffusion-controlled systems with different geometries [108]. By modifying the drug’s diffusivity, the polymer components of hydrogel nanoparticles can be engineered to change the drug’s release kinetics. In addition, regulating the size and shape of hydrogel nanoparticles or altering the mesh size inside the hydrogel matrix can have an impact on the sustainability of drug release [109]. The mesh size is indeed a factor that determines the mechanical strength, diffusivity, and degradability of hydrogel-based systems [110, 111].

It is possible to modify the rate of drug release by designing the chemical reactions that occur within the hydrogel matrix in addition to changing the physical characteristics of a hydrogel. In this case, by adjusting the balance of temperature-dependent interactions between polymer chains, such as hydrophobic contacts and physical entanglements [112], the gelation property of a hydrogel can be adjusted, making the system temperature-sensitive, by incorporating either acidic or basic moieties into the polymer constituents [113, 114], pH-sensitive hydrogel-based systems can also be created. Biomolecule-sensitive hydrogel-based systems that undergo a conformational change in response to changes in the concentration of surrounding biomolecules have been developed using this pH-sensitivity [115]. The glucose-sensitive hydrogel, which combines pH-sensitive moieties with glucose oxidase, is an instance. When glucose permeates the hydrogel, it oxidizes to form gluconic acid. Reduced pH causes the system’s amine functions to get protonated, which causes the hydrogel to swell and release encapsulated bioactive substances, such as chemical medicines and insulin [116, 117]. In fact, in vivo studies have demonstrated the advantages of incorporating stimuli-sensitivity into nanoparticulate systems. The angiopep-2-modified electroresponsive hydrogel nanoparticles serve as an illustration [118]. Because of the electroresponsiveness of the nanoparticles, the release of nonprotein-bound phenytoin sodium (PHT) is triggered when epileptiform activity occurs [118]. In both chemically and electrically generated seizure models, the addition of nanoparticles can enhance the antiseizure properties and lower the effective therapeutic dose of PHT as compared to using PHT alone [118]. This demonstrates how stimuli-responsive carriers might enhance a treatment’s effectiveness. Salt-responsive Alg-based hydrogel nanoparticles, which were created as a protein drug carrier, offer another illustration [119]. These nanoparticles are created by initially interacting Alg with calcium ions under constant stirring to form pre-gel nuclei. Subsequently, these nuclei are stabilized into hydrogel nanoparticles upon the addition of PEI or PEI-graft-polysorbate (PEIP) [119]. Because the nanoparticle structure is responsive to the ionic strength of the surrounding environment, the release of proteins is induced in normal saline but is negligible in distilled water [119]. The observed sensitivity of the nanoparticle structure to microions carries promising implications for drug delivery applications. Specifically, the ability to destabilize the nanoparticle architecture in response to ionic concentrations suggests fine-tuned control over the initiation of drug release. This property allows for release to be triggered precisely when nanoparticles reach the ionic milieu of the bloodstream during clinical administration [100].

BBB plays a crucial role in maintaining balance in the brain, but it poses a significant challenge for delivering drugs to tumor tissues. Tight junctions formed between endothelial cells limit passive diffusion through the extracellular matrix. Consequently, drug transport often relies on transcellular mechanisms, making lipid-soluble drugs more favorable. However, as drugs become more lipid-soluble, they face increased exposure to active efflux mechanisms. Doxorubicin, a lipophilic drug, struggles with poor BBB permeability, primarily due to the active efflux mechanisms found in the BBB membranes [120]. Furthermore, an increase in lipid solubility has been associated with higher drug accumulation at sites other than the intended target [121]. Another obstacle to drug effectiveness within the BBB is the potential degradation of drugs. The cerebral endothelial membrane, abundant in mitochondria, exposes passing solutes to degrading enzymes, such as neprilysin, enkephalin, and insulin-degrading enzymes [122].

Cancer-fighting drugs encapsulated in various nanostructures, incorporated within a larger noninjectable hydrogel, can be specifically administered to brain tumors through implantation. On the other hand, hydrogels with shear-thinning properties are suitable for injection using a syringe [123–125]. Moreover, hydrogel nanoparticles equipped with targeting agents can be introduced intravenously to transport the encapsulated drugs directly to designated brain tumor locations [126].

These filaments containing drugs have a consistently high drug loading, and this loading is adjustable and accurately determined by their molecular design [127].

### In vitro and in vivo drug delivery studies with hydrogel-based nanoparticles, and the impact of drug loading and release on the efficacy of the nanoparticles

Numerous nanoparticles have been investigated for their capability to transport drug payloads into the brain for glioma treatment. While these nanoparticles have undergone in vitro studies, the effectiveness of certain delivery systems has been assessed in vivo through preclinical studies involving animal models, providing real-time efficacy data. Various animal models, including syngeneic, allogeneic, orthotopic xenograft, and genetically engineered models, have been employed to achieve this objective. Syngeneic models, in particular, have emerged as the most commonly utilized due to their low susceptibility to tumor rejection by the immune system [128]. The effectiveness of various agents, including chemotherapeutic drugs, such as temozolomide, doxorubicin, and paclitaxel, as well as miRNA and siRNA, has been evaluated following their delivery through a nanoparticle system. In comparison with alternative delivery methods, nanoparticles have demonstrated superior uptake in the brain and prolonged release of drugs at the tumor site. In addition, the structure of nanoparticles is adaptable, enabling the incorporation of targeting ligands on their surface to enhance precision in targeting the affected tissue [129]. Targeting ligands that exhibit strong binding to receptors overexpressed on the BBB encompass lactoferrin, folic acid, apolipoproteins, and peptides like angiopep-2. In preclinical studies, the utilization of nanoparticles as delivery systems has consistently demonstrated prolonged survival in animals and enhanced drug accumulation in the brain [129, 130]. These studies showcase enhanced anti-tumor drug activity and remarkable regression of tumors [130]. Some of these studies even suggest a potential decrease in the toxic effects of the drug in non-target tissues. Despite the promising findings in preclinical studies, there has been limited success in translating these results to clinical trials.

In laboratory tests, these nanoparticles demonstrate efficient release of DOX molecules upon exposure to laser irradiation. Conversely, in the absence of light, the anticancer agent is securely contained within the particles. In addition, both blank nanogels and loaded nanogels, without light exposure, show minimal toxicity in C6 glioma cells. In contrast, irradiated DOX-loaded hydrogel particles significantly reduce cell viability to a greater extent than free DOX. This heightened efficacy may be attributed to the extensive uptake of these nanostructured systems compared to free DOX. To validate the effectiveness of the designed nanogels, C6 tumor-bearing rats received tail vein injections. Despite widespread distribution among major organs, the nanoparticles concentrated primarily in the tumor. Importantly, the absence of laser irradiation in healthy organs prevented the release of DOX, avoiding undesired effects. In the tumor, the on-demand release of DOX resulted in an impressive 91% reduction in tumor volume, highlighting these nanogels as a potential and effective therapeutic approach against brain cancer [41].

An effective approach involves utilizing Coomassie Brilliant Blue *G*-250 (CB), a visible contrast enhancer, in polyacrylamide-based nanoparticles crosslinked with glycerol dimethacrylate. Three distinct systems were developed using various production methods: CB covalently linked, CB-encapsulated, and CB-post loaded nanoparticles [131].

In vivo studies provide insights into mechanisms that are challenging to replicate in vitro due to the intricate interactions within multicellular and microenvironmental contexts. The integration of the BBB and cerebrovascular structures with the brain in vivo is complex and challenging to accurately mimic in vitro. Moreover, the brain is interconnected with various bodily systems that influence drug and nutrient absorption, circulation, metabolism, and waste excretion. Animal studies adopt a post-resection treatment approach, where a hydrogel is intracerebrally or intracerebroventricularly injected after the removal of a brain tumor. This hydrogel may be loaded with an anti-cancer drug to facilitate sustained release. Administering through this route enables the material to be placed at the target site without significant hindrance, as the BBB acts as a physicochemical barrier to substances [132] Puente et al. conducted an experiment involving the injection of a hydrogel loaded with TMZ (Temozolomide) and 131I into the surgical cavity of the mouse brain post-resection. The semisolid nature of the hydrogel provided a balance, being neither too rigid nor releasing TMZ too rapidly. Consequently, it efficiently distributed the drug, leading to enhanced survival among the treated animal subjects. The use of hydrogels containing chemotherapy-loaded particles demonstrated improved survival compared to untreated groups, attributed to the sustained release of the drug [133–135]. In addition, these hydrogels have the capability to encapsulate radioactive isotopes, facilitating simultaneous radiotherapy [132]. Administering hydrogels postoperatively serves to prevent the recurrence of brain tumors. This is achieved by utilizing nano- or microstructures loaded with drugs, which effectively restrict toxicity to areas outside the target site, where the hydrogel is injected [136]. Specific formulations of hydrogels can be monitored in real-time using imaging techniques, such as the visualization of incorporated iron oxide magnetic nanoparticles [137]. Theranostic hydrogels offer substantial advantages when evaluated in vivo, serving dual purposes for both treating the condition and monitoring the patient’s status [138].

## Potential toxicity of nanoparticles on humans

Nanoparticles, including hydrogel-based nanoparticles, have gained substantial interest for their biomedical applications, such as drug delivery, imaging, and diagnostics. Despite their promising utility, the unique physicochemical properties of nanoparticles also pose potential risks to human health, necessitating a comprehensive understanding of their toxicity [139].

### Cellular uptake and cytotoxicity

Nanoparticles can enter cells via various pathways, including endocytosis and passive diffusion. Once inside, they can localize in different cellular compartments, such as lysosomes, mitochondria, and the nucleus. This intracellular presence can disrupt normal cellular functions, leading to cytotoxic effects [140].

- **Oxidative stress**: Many nanoparticles can generate reactive oxygen species (ROS), leading to oxidative stress. This stress can damage cellular components, including lipids, proteins, and DNA, potentially triggering apoptosis or necrosis. For instance, studies have demonstrated that silver nanoparticles can induce significant oxidative stress and cytotoxicity in lung epithelial cells [141, 142].
- **Inflammation**: Nanoparticles can activate inflammatory pathways, resulting in the release of pro-inflammatory cytokines. This inflammation can lead to chronic conditions if exposure is prolonged. Titanium dioxide nanoparticles, for example, have been shown to induce inflammation in both in vitro and in vivo models [143, 144].

### Immune system interaction

The interaction of nanoparticles with the immune system is complex and can lead to either suppression or over-activation of immune responses.

- **Immunogenicity**: Nanoparticles can be recognized as foreign entities by the immune system, leading to their uptake by macrophages and dendritic cells. This recognition can result in the activation of innate and adaptive immune responses, which might cause inflammation or autoimmunity [145]. The surface properties of nanoparticles, such as charge and functional groups, play a significant role in determining their immunogenicity [146].
- **Allergic reactions**: Certain nanoparticles can provoke allergic reactions, particularly in individuals with pre-existing sensitivities. For instance, carbon nanotubes have been found to exacerbate allergic airway inflammation in mouse models [147, 148].

### Organ accumulation and long-term effects

After entering the bloodstream, nanoparticles can be distributed to various organs, where they might accumulate and exert toxic effects over time.

- **Liver and spleen**: These organs are primary sites for nanoparticle accumulation due to their role in filtering blood. Persistent nanoparticle presence can lead to hepatic and splenic toxicity, characterized by inflammation, fibrosis, and functional impairments [149].
- **Lungs and brain**: Inhalation of nanoparticles can lead to pulmonary toxicity, with potential consequences, such as chronic obstructive pulmonary disease (COPD) and lung cancer. In addition, nanoparticles can cross the blood–brain barrier, potentially leading to neurotoxicity, which manifests as behavioral changes and cognitive impairments [150].

### Genotoxicity and carcinogenicity

The genotoxic potential of nanoparticles is a significant concern, as it can lead to mutations and cancer development.

- **DNA damage**: Nanoparticles can directly interact with DNA or indirectly induce damage through oxidative stress. This interaction can cause mutations, chromosomal aberrations, and other genotoxic effects. For instance, metal oxide nanoparticles such as zinc oxide and titanium dioxide have been reported to induce DNA strand breaks and chromosomal instability in mammalian cells [151].
- **Carcinogenicity**: Long-term exposure to certain nanoparticles has been associated with the development of cancer. For example, studies have shown that chronic exposure to carbon nanotubes can lead to mesothelioma, a type of cancer affecting the lining of the lungs [152].

### Biodegradability and clearance

The biodegradability and clearance of nanoparticles are critical determinants of their safety profile.

- **Biodegradable nanoparticles**: Ideally, nanoparticles should be designed to degrade into non-toxic components that can be easily eliminated from the body. For instance, hydrogel-based nanoparticles made from biodegradable polymers such as PLGA (poly(lactic-co-glycolic acid)) are considered safer due to their ability to degrade into lactic and glycolic acid, which are naturally metabolized by the body [153].
- **Non-biodegradable nanoparticles**: Non-biodegradable nanoparticles, such as certain metal or carbon-based nanoparticles, pose a higher risk due to their persistence in the body. Prolonged retention can lead to chronic toxicity and accumulation in vital organs, raising concerns about long-term health effects [154].

## Imaging and tracking of hydrogel-based nanoparticles

### Techniques for imaging and tracking hydrogel-based nanoparticles in vivo, such as magnetic resonance imaging (MRI), fluorescence imaging, and positron emission tomography (PET)

Various modalities are employed for biomedical imaging applications, but only a subset is extensively used for hydrogel imaging. Taking into account factors, such as penetration depth, image resolution, contrast source, and imaging objectives, the most relevant modalities include CT, MRI, fluorescence imaging. In addition, there are less commonly used modalities, such as nuclear imaging, photoacoustic imaging, and ultrasound [155].

In in vivo studies involving rats with C6 glioma tumors, the use of Cy5.5-Lf-MPNA nanogels showed a growing fluorescence signal that correlated with the iron concentration. This observation indicates that Cy5.5-Lf-MPNA nanogels can effectively target glioblastoma multiforme (GBM) through both passive and active strategies. Furthermore, these nanogels have the capability to identify and delineate the margins of GBM tumors, owing to their established MR/fluorescence imaging abilities [156].

In addition to delivering chemical drugs, hydrogel nanoparticles can also be loaded with contrast agents for complementary imaging. An earlier in vivo study showcased this capability, where polyacrylamide hydrogel nanoparticles, covalently linked with Coomassie Blue molecules, were intravenously administered to a rat brain tumor model once the tumor radius reached 1–2 mm [131]. The outcomes indicated that the nanoparticles, with their tumor-specific visible color staining, can facilitate real-time color-guided tumor resection. This process eliminates the need for special lighting conditions or equipment, providing a practical approach for intraoperative tumor margin delineation during brain cancer surgery [131]. Moreover, the surface of the nanoparticles has been modified by conjugating PEG and F3 peptides. This modification has improved the targeting specificity towards tumors and extended the circulation time of the nanoparticles in the bloodstream [131].

Like other imaging modalities mentioned previously, fluorescent hydrogels have been investigated for monitoring implants and various theranostic applications. Specifically, hydrogels conjugated with upconversion nanoparticles (UCNPs) have been documented for the extended in vivo tracking of the distribution and degradation of hydrogels [157]. In addition to their fluorescent properties, UCNPs can serve in photodynamic therapy (PDT) and photothermal therapy (PTT) for cancer. UCNPs have the capability to activate surrounding photosensitizer molecules, inducing the generation of reactive oxygen species and heat, which can be harnessed to eliminate tumor cells [158]. The amalgamation of UCNPs and hydrogels can function not only as tumor imaging probes but also as therapeutic agents. For example, gelatin hydrogels loaded with doxorubicin and UCNPs were employed for chemophotothermal therapy, exhibiting anti-tumor effects, along with upconversion fluorescence imaging capabilities [159]. Likewise, a hybrid system was created, combining an injectable silk fibroin nanofiber hydrogel for tumor upconversion luminescence imaging and photothermal therapy [160]. In addition to UCNPs, fluorophores can also be employed to impart fluorescent properties to hydrogels. Park et al. designed fluorescent hydrogels using hyaluronic acid (HA) and gelatin by attaching an 800 nm indocyanine near-infrared (NIR) fluorophore, ZW800-3a, through its carboxylic functional group to the amine groups in gelatin [161]. The researchers were able to concurrently track scaffold degradation and brain tissue regeneration by imaging the hydrogel using the 800 nm channel and observing brain tissue ingrowth with the 700 nm channel, employing a 700 nm active brain-specific contrast agent. In addition, other studies have demonstrated the integration of various fluorescent probes into hydrogels for comparable applications in hydrogel tracking, drug delivery, and fluorescence-guided surgery [107, 162, 163].

In the context of tumor resection, the precise identification of neoplastic cells is crucial for optimizing surgical outcomes. Hydrogel nanoparticles, given their small size and capability for tumor infiltration, present a potentially effective approach for actively targeting and identifying glioblastoma multiforme (GBM) cells. With the goal of enhancing tumor visualization, Jiang L. et al. explored this avenue [156]. They developed a system using Fe3O4 nanoparticles loaded poly(N-isopropylacrylamide-co-acrylic acid) (MPNA) nanogels. The magnetic nanogel exhibited inherent pH/temperature sensitivity, and further conjugation with Cyanine5.5 NHS (Cy5.5)-labeled lactoferrin (Lf) introduced a targeted contrast agent for preoperative MRI and intraoperative fluorescence imaging of tumors. In vitro studies involved two cell lines: C6 glioma cells, expressing high levels of low-density lipoprotein receptor-related protein 1 (LRP1), a known Lf receptor; and ECV 304 cells with no LRP1 expression. As expected, both Cy5.5-Lf-MPNA nanogels and Cy5.5-Lf-Fe3O4 nanoparticles exhibited high internalization by C6 cells, compared to MPNA nanogels and Fe3O4, respectively. This trend did not occur with ECV 304 cells, as there were no significant differences among formulations. Interestingly, cellular uptake at pH 6.8 was higher than at pH 7.4 for both C6 and ECV 304 cells. This is attributed to the hydrophilic and enlarged state of Cy5.5-Lf-MPNA nanogels at pH 7.4, enhancing blood circulation time, and their conversion to a hydrophobic state with smaller size at pH 6.8 (tumor microenvironment), favoring internalization by GBM cells. In vivo studies with rats bearing C6 glioma tumors confirmed these findings, as Cy5.5-Lf-MPNA nanogels exhibited increasing fluorescence signals proportionate to the iron concentration. The study demonstrated that Cy5.5-Lf-MPNA nanogels can actively target GBM cells through both passive and active strategies while also identifying and outlining GBM tumor margins due to their established MR/fluorescence imaging capabilities [156].

### Applications of imaging and tracking in monitoring treatment efficacy, and the potential of these techniques to provide information on the pharmacokinetics and biodistribution of the nanoparticles

A novel cisplatin-loaded nanogel with surface modification was created in light of the overexpression of membrane proteins connexin 43 (Cx43) and brain-specific anion transporter 1 (BSAT1) in glioma and peritumoral cells. PEG-b-poly (methacrylic acid) and MAL–PEG–NH2 were used as starting ingredients to create the hydrogel nanoparticles, which were then conjugated with CIS and antibodies. After the nanogels were deloaded, their diameter was 120–130 nm, their polydispersity index was 0.13, and their zeta potential was − 15 ± 5 mV. These results suggested that the nanogels could be laden with CIS, with a capacity of 30–35% and an entrapment efficiency of 45% [102]. Moreover, after a week, about half of the CIS was released from these particles, indicating persistent release over time. Comparing these nanogels to free CIS, cellular research revealed that they were less toxic to C6 cells. On the other hand, in vivo findings showed that rats treated with these nanoparticles and implanted with glioma 101/8 cells had an improved overall survival rate. It is expected that the antibody–receptor interactions will target the hydrogel nanoparticles and help maintain high levels of CIS close to the tumor [102].

In vivo results from mice with subcutaneously induced U87 MG tumors revealed that treatment with these hydrogel nanoparticles successfully inhibited tumor growth, a result not achieved with unmodulated nanogels and free doxorubicin (DOX). In addition, the lower incidence of adverse effects indicates the potential of this platform as a novel approach against glioblastoma multiforme (GBM). In a related development, alginate nanogels co-loaded with gold nanoparticles and cisplatin (CIS) were recently designed to enhance the radiosensitization of cancer cells for radiotherapy treatments. This nanocomplex exhibited higher cytotoxicity against U87 MG cells compared to the free drug. Furthermore, the platform demonstrated a pronounced apoptotic effect after *X*-ray irradiation on the same cell line, showcasing its potential for combined therapeutic strategies [164]. However, additional studies are necessary to establish the in vivo efficacy of this nanocomplex.

An intriguing example involves the utilization of a magnetic resonance imaging (MRI) traceable ultra-thermosensitive hydrogel. This hydrogel is composed of negatively charged carboxymethyl cellulose (CMC)-grafted poly(N-isopropylacrylamide-co-methacrylic acid) (CMC-g-PNIPAAmMA) and positively charged gadopentetic acid/branched polyethylenimine (DTPAGd/bPEI), incorporating epirubicin as a hydrophilic drug (hydrogelGd/EPI). Simultaneously, it integrates bovine serum albumin (BSA) nanoparticles encapsulating paclitaxel as a hydrophobic drug (BSA/PTX). This innovative system is designed for in situ drug delivery or in residual tumor tissues after surgical resection, aiming to prevent disease recurrence [165].

Kim J.I. et al. developed a magnetic resonance imaging (MRI)-monitored long-term therapeutic hydrogel (MLTH) system, combining a thermosensitive/magnetic poly(organophosphazene) (PPZ) hydrogel loaded with PEGylated cobalt ferrite (*P*-CoFe2O4) nanoparticles as an imaging platform and SN-38 (active metabolite of irinotecan) as a chemotherapeutic agent. The hydrogel, containing *P*-CoFe2O4 nanoparticles, was created through hydrophobic interactions, and the final formulation was achieved by physically mixing SN-38 with the magnetic hydrogel. In vitro release assessments indicated that the MLTH platform can sustainably release the drug due to the long-term biodegradation of the hydrogel, monitored by both the polymer concentration and SN-38 amount. When tested in U87 MG tumor-bearing mice over 22 days, the MLTH demonstrated a prolonged inhibition of tumor growth. In summary, this system is designed for injection with a reversible sol–gel phase transition close to body temperature, facilitating sustained drug release and serving as an MR imaging agent. The MRI aspect provides spatial and temporal information regarding the MLTH-treated and untreated areas of glioblastoma multiforme (GBM) in MR images over time [137, 166].

## In vitro studies on hydrogel-based nanoparticles for brain tumors management

Comparing free and hydrogel-loaded micelles in in vitro release experiments revealed that the hydrogel system produced a greater sustained release while also reducing the burst effect. An additional in vitro experiment demonstrated that the hydrogel was fully hydrolyzable in the presence of collagenase, which further promoted paclitaxel (PTX) release. Research using the C6 cell line validated the hydrogel system’s applicability. The hydrogel loaded with micelles was found to be more toxic to cancer cells than PTX alone. This could be attributed to the absorption of micelles by cells and the quick release of free PTX from tumor cells. All things considered, this hydrogel nanocomposite is a promising method to improve the therapeutic effectiveness of PTX in the surgically repaired resection cavity [167]. In a study conducted by Xu Y. et al. [168] the co-administration of paclitaxel (PTX) and temozolomide (TMZ) was designed using a combination of PEG–PLGA nanoparticles and a hydrogel based on PF127. Using a double emulsification/solvent evaporation process, the medicines were concurrently integrated into the nanoparticles, taking into account their respective solubilities. To further modify gelation and rheological characteristics, substances such Pluronic^®^ F68, sodium alginate, and hydroxypropyl methylcellulose were added to PF127 solutions. The release of both drugs was found to be dependent on and controlled by the composite hydrogel corrosion. In vitro studies with U87 MG and C6 cell lines indicated that the gel promoted the most potent growth-inhibiting and apoptosis-inducing effects. For PTX/TMZ solution, PTX/TMZ nanoparticles, and the gel, the apoptosis rates in U87 MG cells were 23.6%, 26.4%, and 32.5%, respectively, while in the C6 cells, they were 26.0%, 30.0%, and 39.2%, respectively [168].

Alginate was employed to encapsulate PLGA–PTX microspheres within a solid hydrogel matrix to mitigate the initial burst effect and regulate the release of the drug from the microcarriers. This hydrogel was meticulously designed and characterized, undergoing in vitro testing for its release pharmacokinetics and cytotoxicity. In vivo studies, particularly in a subcutaneous tumor model, yielded promising results. Furthermore, when evaluated using an intracranial human glioblastoma multiforme (GBM) xenograft model, this hydrogel exhibited a significant inhibition of tumor growth, with the drug penetrating up to 5 mm from the implant site for up to 42 day post-implantation [169, 170]. Ulrich et al. demonstrated that an increase in hydrogel stiffness resulted in enhanced glioma cell proliferation. The observed variation in cell response between 2 and 3D cultures highlights the significance of dimensionality when creating in vitro models to investigate the interactions between glioma cells and niches. Tumor cells need to get beyond the extracellular matrix’s physical restrictions to proliferate and migrate in vivo. In 2D culture, there is no physical restraint; therefore, cells are not exposed to the rises in mechanical stress that come with physical limitation in 3D. To replicate tumor growth in vivo, 3D biodegradable hydrogels with tissue-mimicking biochemical and biophysical signals may provide a more physiologically accurate model [171]. In glioma cells, the inhibition of Rho-associated protein kinase 1 (ROCK1) and 2 has been reported to result in differences in cell cycle progression [172]. In addition, Zohrabian et al. observed that the introduction of a Rho-associated protein kinase (ROCK) inhibitor to glioblastoma cultures in 2D settings resulted in reduced radial migration [173]. The heightened expression of ROCK1 in the stiff hydrogels might elucidate the increase in the number of actin protrusions at the periphery of the spheroid in such hydrogels. Despite the considerable matrix stiffness, the cells could actively reorganize their actin cytoskeleton to explore and invade the matrix. This phenomenon aligns with observations in breast cancer cell lines when subjected to compression in a 2D environment [174]. A drug–peptide analogue with a reverse bolaamphiphile (RBA) design has been created by researchers. It has two separate hydrophobic terminals and a central hydrophilic area that contains an enzyme-responsive section that binds to matrix metalloproteinase-2 (MMP-2). To enable enzymatically triggered hydrogel breakdown, this RBA design is essential for exposing the enzyme–substrate on the surface of the formed filament structures. A conventional peptide amphiphile design, on the other hand, did not show an MMP-2 response. The drug-based RBA molecule had similar toxicities to the free drug in in vitro applications. Furthermore, in a three-dimensional context, faster hydrogel disintegration and more efficient cancer cell killing were attributed to increased MMP levels in denser cell environments. This RBA design is intended for local tumor treatment, specifically following intratumoral administration or surgical excision, according to the researchers. By allowing the hydrogel to display a drug release profile that matches the size and aggressiveness of the surrounding tumor, the MMP responsiveness characteristic would improve overall efficacy [127].

## In vivo studies on hydrogel-based nanoparticles for brain tumors management

Studies conducted in vitro show that medications are efficient in causing brain tumor cells to undergo apoptosis. However, contradictory in vivo outcomes have been found because of the obstacles related to traditional administration [175]. Compared to alternative local drug delivery systems, a further benefit of using a starch-based hydrogel is that the matrix may be constructed with pharmaceuticals encapsulated and can be injected safely and easily, as shown in prior in vivo investigations [176]. Two distinct models were used for the in vivo studies: MBR 614 tumor-bearing mice and human glioma U87 MG tumor-bearing animals. In comparison with the control group, free EPI, and unloaded BSA nanoparticles incorporated in the hydrogel, findings showed that the implantation of BSA/PTX nanoparticles incorporated in hydrogelGd/EPI led to a substantial rise in average survival, efficient tumor reduction, and prevention of relapse [177]. For the release of camptothecin (CPT), Shah S. et al. [178] created an attractive photo-triggerable hybrid platform made of silica nanoparticles enclosed in a PEG-based hydrogel. Human U87 MG cells harboring a mutant version of the epidermal growth factor receptor vIII (EGFRvIII) were used to test this platform. A photo-triggerable chemical adaptor was synthesized using 4-hydroxymandelic acid and then covalently bound to CPT. This complex was attached to the surface of silica nanoparticles and subsequently encapsulated within the hydrogel matrix. It was anticipated that the chemical adapter would become active in response to photoirradiation, releasing the covalently bound medication from the PEG-based hydrogel. This phenomena was verified using a GBM cell line, showing that cells exposed to UV radiation had much lower viability than those that were not [178].

**Fig. 2:**
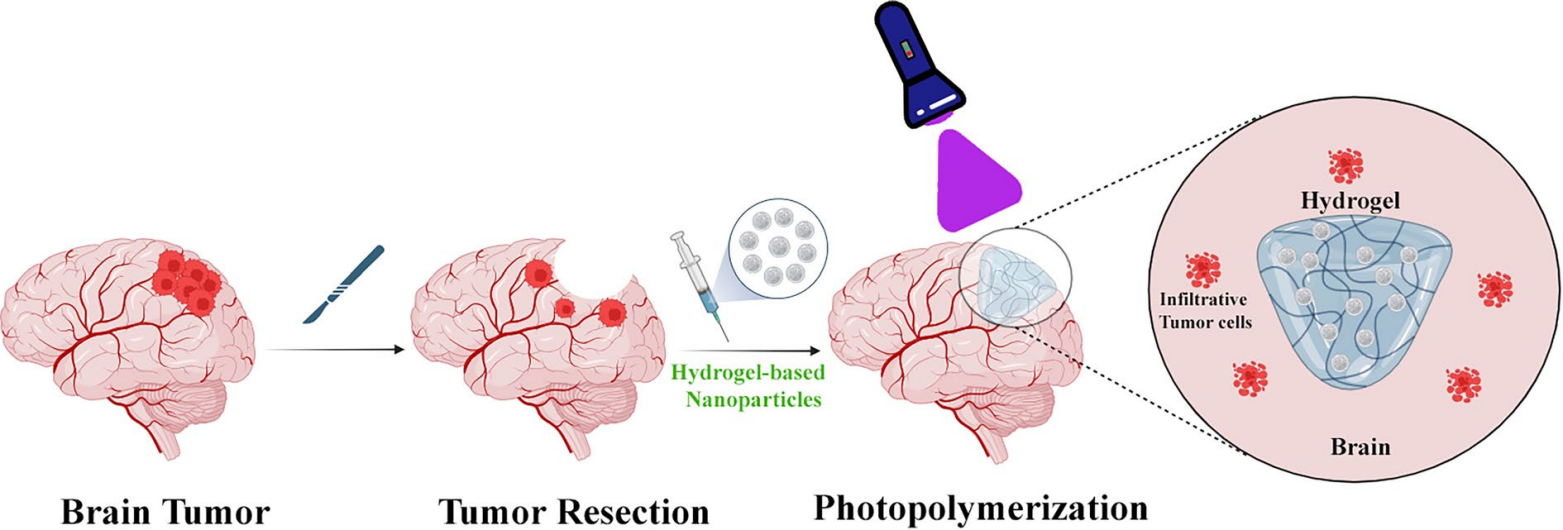
A Schematic representation of the codelivery of anticancer drug for instance PTX and TMZ through a photopolymerizable hydrogel for the postresection treatment of glioblastoma. It shows how a brain tumor is surgically removed and treated with a hydrogel containing PTX and TMZ-loaded nanoparticles, which is solidified by UV light and releases the drugs to target the remaining tumor cells in the brain tissue

Others have reported on the feasibility, efficacy, and tolerability of a hydrogel composed of lipid nanocapsules loaded with lauroyl-gemcitabine (GemC12-LNC), an amphiphilic derivative of gemcitabine, for the local treatment of GBM [179, 180]. This injectable formulation consists solely of lipid nanocapsules and the cytotoxic drug. It is prepared using a cost-effective and solvent-free method, employing components approved by the US Food and Drug Administration (FDA) [180]. The mechanical properties of this hydrogel are tailored for brain implantation, and its degradation aligns with the sustained release of the drug. In vitro studies demonstrate that the drug release persists for over 1 month [180]. In vivo, this system is well tolerated in the mouse brain and demonstrates a reduction in tumor growth in a murine orthotopic human xenograft GBM model following intratumoral administration [179]. They devised a straightforward and dependable resection technique utilizing a *U*-87 MG xenograft model in nude mice. This technique involves the use of a biopsy punch to cut the brain region containing the tumor [181]. This procedure proved successful in demonstrating the capacity of the GemC12–LNC hydrogel to impede the formation of recurrences [179]. A photopolymerizable poly (ethylene glycol) dimethacrylate (PEG–DMA)-based hydrogel was developed for local delivery of temozolomide (TMZ) into the brain. The initial step involved the preparation of TMZ-loaded PEG750-(Poly("ε-caprolactone-co-trimethylene carbonate)) polymeric micelles (M-TMZ) to enhance solubilization of the hydrophobic drug. Subsequently, M-TMZ-loaded hydrogels (M-TMZ/PEG-DMA) were formed, designed for injection and rapid photopolymerization in glioblastoma multiforme (GBM) resection cavities using UV light (Fig. 2). The TMZ release profile resembled that of the reference Gliadel^®^, with a 45% burst release in the first 24 h and a logarithmic release of 20% over the initial week. In addition, the system exhibited robust in vivo antitumor efficacy, characterized by significant apoptosis and reduction of tumor mass in xenograft U87 MG tumor-bearing mice [182]. Results from studies in subcutaneously induced U87 MG tumor-bearing mice revealed that treatment with these hydrogel nanoparticles successfully inhibited tumor growth. In contrast, unmodulated nanogels and free doxorubicin (DOX) were unable to prevent this outcome. In addition, the lower occurrence of adverse effects suggests the potential of this platform as a new and promising approach against GBM [41].

## Challenges and limitations of hydrogel-based nanoparticles for brain tumors management

Indeed, while hydrogel-based nanoparticles present promising strategies for managing brain tumors, they encounter significant challenges and limitations:

Physiological Barriers: One of the primary challenges lies in overcoming physiological barriers, particularly the blood–brain barrier. The BBB serves as a protective barrier, restricting the penetration of therapeutic agents into the brain. While designed to shield the brain from harmful substances, it simultaneously poses a significant obstacle for nanoparticle-based treatments, limiting their effectiveness in reaching and treating brain tumors. Overcoming these physiological barriers remains a critical focus in the development of effective hydrogel-based nanoparticle therapies for brain tumors [183]. Recent advancements have focused on creating hydrogels intended for localized drug delivery, which helps to reduce systemic exposure. This method is crucial for reducing the adverse effects typically associated with conventional chemotherapy [184].

Furthermore, physiological barriers, including the immune response, can influence the efficacy of hydrogel-based nanoparticles. The immune system might identify these nanoparticles as foreign entities, resulting in quick removal from the bloodstream and diminished therapeutic efficacy. Efforts are underway to enhance the biocompatibility of these systems, including the exploration of surface modifications using biocompatible polymers to address this challenge [185, 186].

Complex Resistance Mechanisms: Brain tumors frequently manifest intricate drug resistance mechanisms, contributing to high rates of treatment failure. Among these mechanisms are efflux pumps within tumor cells that actively expel drugs, as well as genetic mutations that render tumor cells less responsive to chemotherapy. Efflux pumps play a crucial role in multidrug resistance (MDR) by reducing the intracellular concentrations of therapeutic agents. This phenomenon presents important issues regarding GBM, as the tumor microenvironment could potentially exacerbate drug resistance. Hydrogel-based nanoparticles need to be designed to either block these efflux mechanisms or to deliver drugs in a manner that avoids them, potentially through the use of combination therapies that incorporate efflux pump inhibitors alongside standard chemotherapeutics [187].

Furthermore, the genetic diversity of brain tumors introduces an additional level of complexity. Tumor cells can possess mutations that provide resistance to certain medications, making conventional therapies ineffective. For example, changes in signaling pathways may result in decreased sensitivity to alkylating agents, such as temozolomide, which is frequently utilized in the treatment of GBM [188].

These complex resistance mechanisms pose formidable challenges for the effectiveness of hydrogel-based nanoparticle therapies, necessitating innovative approaches to overcome or circumvent these resistance pathways in the pursuit of more successful brain tumor management [189].

Limited Therapeutic Window: Nanoparticle-based systems for brain tumor management face a restricted therapeutic window owing to the presence of physiological barriers and resistance mechanisms. Striking the delicate balance between efficacy and safety becomes a significant challenge. The therapeutic window refers to the range of drug concentrations, where therapeutic benefits are maximized, and adverse effects are minimized. Overcoming this limitation requires precise fine-tuning of nanoparticle formulations and delivery strategies to optimize therapeutic outcomes while mitigating potential risks and side effects [190].

Delivery and Targeting: A significant hurdle in hydrogel-based nanoparticle applications is ensuring efficient targeting of tumor cells without impacting healthy cells. Although nanogels can be engineered to possess both loading and targeting properties, achieving precise delivery to tumor sites remains a complex task. Overcoming this challenge involves the development of sophisticated delivery systems that can navigate the intricate biological environment and selectively target cancer cells while sparing healthy tissues. Enhancing the specificity and efficacy of delivery mechanisms is crucial for maximizing the therapeutic impact of hydrogel-based nanoparticles in brain tumor management [41].

Researchers have concentrated on developing nanogels and hydrogels that possess improved targeting abilities and mechanisms for controlled release to tackle these challenges. A promising strategy involves the creation of multifunctional hydrogels capable of concurrently delivering chemotherapeutic agents while also modulating the immune response. Wang et al. presented a zwitterionic injectable hydrogel that integrates chemotherapy with immunotherapy, successfully inhibiting tumor recurrence after surgery by boosting the cytotoxic *T* cell response and reducing the activity of regulatory *T* cells. This dual-action approach emphasizes the capability of hydrogels to serve both as drug delivery systems and as agents that stimulate the immune system to more effectively target cancer cells [191].

Ongoing Research and Development: Despite the challenges outlined, continuous research and development efforts are dedicated to enhancing the design and functionality of hydrogel-based nanoparticles to improve their performance in brain tumor management.

Recent research has concentrated on diverse strategies, such as the implementation of targeted delivery systems, multifunctional nanoparticles, and innovative materials to address these challenges.

A notable development pertains to the application of dual-targeting ligands on nanoparticles, aimed at improving their specificity for glioma cells. For example, Li et al. illustrated the efficacy of dual peptide-modified liposomes, which were engineered to specifically target glioma cells through the attachment of angiopep-2 and tLyP-1 peptides. This method not only enhanced targeting efficiency but also aided in the delivery of therapeutic agents into the tumor microenvironment, thus improving the therapeutic effectiveness of docetaxel in the treatment of glioma [7].

Another promising approach involves the use of nanoparticles that can influence the tumor microenvironment to enhance drug delivery. Feng et al. emphasized the possibility of augmenting blood flow in tumor regions, which may improve the efficiency of nanoparticle delivery. This approach holds significant importance in the realm of glioblastomas, where irregular blood vessel formation frequently obstructs efficient drug delivery [192].

### Synthesis and Characterization Challenges:

The synthesis and characterization of hydrogel-based nanoparticles involve complex processes with several technical challenges that can impact their development and practical application. Here’s a comprehensive overview of these challenges:

*Stability Issues:* Hydrogel nanoparticles often encounter stability issues, especially when exposed to physiological conditions. Premature swelling or degradation may occur, leading to the premature release of encapsulated drugs or bioactive molecules before reaching the target site. Ensuring long-term stability in various environments is critical for their effectiveness as drug delivery systems. Addressing these stability challenges is vital for advancing the practical application of hydrogel-based nanoparticles in clinical settings [193]. Reproducibility: Consistent and reproducible synthesis of hydrogel nanoparticles poses a challenge due to the sensitivity of the polymerization process to factors, such as temperature, pH, and initiator concentration. Variations in these parameters can result in significant differences in nanoparticle size, shape, and functionality. Overcoming these challenges requires meticulous control of synthesis conditions and optimization of protocols to ensure the reliable production of hydrogel nanoparticles with consistent characteristics. Enhancing reproducibility is crucial for the scalability and practical application of these nanoparticles in medical settings [194].

*Scalability:* Scaling up the production of hydrogel nanoparticles from laboratory to industrial scale presents significant challenges. Conditions optimized for small-scale synthesis may not directly translate to larger volumes, potentially affecting the quality and uniformity of the nanoparticles. Achieving scalability requires careful consideration of factors, such as reaction kinetics, mass transfer, and overall process engineering. Overcoming these challenges is essential for the successful translation of hydrogel-based nanoparticles from research and development stages to large-scale production, ensuring their viability for widespread medical applications [195].

*High Costs:* The synthesis of hydrogel nanoparticles can incur high costs due to several factors. Specialized equipment, high-purity monomers, and cross-linkers, along with the requirement for controlled environments, contribute to the overall expenses of production. Addressing the high costs associated with the materials and methods used in synthesis is crucial for making hydrogel-based nanoparticles more accessible and feasible for widespread use in medical applications. Efforts to optimize production processes and explore cost-effective alternatives are essential to mitigate economic barriers and enhance the affordability of these advanced nanomaterials [196].

*Characterization Limitations:* Characterizing the complex structure of hydrogel nanoparticles poses challenges, as it demands advanced and often costly analytical techniques. Accurately determining properties such as particle size distribution, surface charge, and mechanical strength can be challenging and time-consuming. Overcoming these characterization limitations involves continued advancements in analytical tools and methodologies, as well as the development of more efficient and cost-effective characterization techniques. Improving the accuracy and accessibility of nanoparticle characterization is pivotal for understanding and optimizing the properties of hydrogel-based nanoparticles in a variety of medical applications [197].

*Biocompatibility and Toxicity:* Ensuring the biocompatibility and minimizing the potential toxicity of hydrogel nanoparticles is essential for their use in medical applications. Comprehensive biological testing is required to assess their safety, and this process can be both lengthy and complex. Addressing biocompatibility and toxicity concerns involves thorough testing across various biological systems to understand the impact of hydrogel nanoparticles on living tissues. Striking a balance between effective therapeutic delivery and minimal adverse effects is a critical consideration in advancing the development and clinical application of hydrogel-based nanoparticles [198].

**Addressing challenges and innovations:** Researchers are actively engaged in overcoming the challenges associated with hydrogel-based nanoparticles. Their efforts include:

1. **New synthesis methods:** Developing novel synthesis methods to enhance the reproducibility and scalability of hydrogel nanoparticles.
2. **Improved characterization techniques:** Advancing characterization techniques to accurately assess properties, such as particle size distribution, surface charge, and mechanical strength.
3. **Cost reduction:** Exploring alternative materials and optimizing production processes to reduce costs associated with synthesis.

These endeavors collectively aim to enhance the stability, reproducibility, and cost-effectiveness of hydrogel-based nanoparticles, making them more practical for medical applications.

**Biological challenges:** The biological challenges in targeting and delivering drugs to brain tumor cells are multifaceted. Two major barriers include:

**Blood–brain barrier (BBB):** A highly selective permeability barrier that protects the brain from foreign substances. However, it significantly hinders the delivery of therapeutic agents to brain tumors. Overcoming this barrier is crucial for effective brain tumor treatment [199]. The BBB’s tight junctions and efflux pumps actively prevent many drugs from reaching the brain tissue. This necessitates the development of novel drug delivery systems capable of bypassing or penetrating this barrier. Overcoming the challenges posed by the BBB is a critical step in ensuring the effective delivery of therapeutic agents to brain tumors, and it requires innovative solutions to enhance the permeability of the barrier for targeted treatments [200] (Fig. 1).

**High interstitial pressure:** Brain tumors frequently display elevated interstitial pressure, creating a physical barrier to drug penetration. This heightened pressure can lead to a reduction in drug convection through the tumor tissue, posing a challenge for therapeutic agents to effectively reach and treat all areas of the tumor. Addressing the impact of high interstitial pressure is crucial for optimizing drug delivery systems and ensuring comprehensive treatment coverage within brain tumors. Innovative strategies are required to overcome this physical barrier and enhance the uniform distribution of therapeutic agents throughout the tumor microenvironment [201].

The biological challenges presented by the BBB and high interstitial pressure in brain tumors underscore the need for concerted efforts in developing targeted drug delivery systems. These systems must navigate the intricate brain environment, delivering effective treatments to tumor cells while minimizing harm to healthy brain tissue. Innovations in drug delivery technologies are essential for overcoming these biological barriers and improving the precision and efficacy of brain tumor treatments.

## Future directions for hydrogel-based nanoparticles in brain tumors management

Future hydrogel systems are poised to deliver drugs directly to the tumor site, introducing a high degree of specificity and minimizing systemic side effects. This localized approach is anticipated to significantly enhance the overall efficacy of brain tumor treatments, providing a more precise and targeted therapeutic strategy [202, 203].

**Nanogels:** Nanogels are garnering attention for their systemic administration potential and precise targeting of tumor cells. These diminutive yet potent agents can carry therapeutic payloads directly to cancer cells, representing a substantial advancement in brain tumor management. The development of nanogels holds promise for refining treatment strategies and improving the delivery of therapeutic agents to brain tumors with increased precision [41].

**Engineered hydrogels:** Progress in engineered hydrogels is directed towards establishing more accurate in vitro models of brain tumors. This advancement offers a platform for enhanced comprehension of tumor behavior and facilitates the testing of novel treatments. Improved in vitro models contribute to a deeper understanding of brain tumor dynamics and provide valuable insights for the development and evaluation of innovative therapeutic interventions [90].

**Hybrid systems:** The exploration of integrating hydrogels with other therapeutic modalities, such as phototherapy and magnetic stimulation, is a promising avenue to enhance treatment outcomes. These hybrid systems have the potential to provide a multifaceted approach to treating brain tumors, leveraging the synergies between different therapeutic strategies for improved efficacy. The integration of diverse therapeutic modalities within hydrogel systems represents an innovative direction for advancing the treatment landscape for brain tumors [204].

**Computational modeling:** The growing significance of computational studies in predicting the behavior of hydrogel-based nanoparticles is noteworthy. This approach plays a crucial role in optimizing the design of these nanoparticles for more effective brain tumor management. By leveraging computational modeling, researchers can gain insights into the intricate interactions and dynamics of hydrogel-based systems, contributing to the refinement and enhancement of their performance in brain tumor treatments [205].

### Advancements in hydrogel-based nanoparticles

#### Synthesis and characterization

**Biodegradable polymers:** The incorporation of biodegradable polymers, including polylactic acid (PLA), polyglycolic acid (PGA), and polycaprolactone (PCL), in the synthesis of hydrogel-based nanoparticles is gaining momentum. These polymers possess the advantage of degrading into non-toxic byproducts that can be easily eliminated from the body. This property is especially advantageous for drug delivery systems, contributing to the reduction of long-term side effects [207].

**Smart hydrogels:** The integration of smart hydrogels represents a notable advancement. Engineered to respond to stimuli, such as pH, temperature, and biological signals, these materials exhibit the ability to dynamically change their properties in response to the tumor microenvironment. This characteristic facilitates targeted drug release, contributing to enhanced treatment efficacy and personalized therapeutic approaches. Smart hydrogels hold promise for refining the precision and adaptability of hydrogel-based nanoparticles in the realm of brain tumor management [206].

New developments in hydrogel-based nanoparticles have significantly impacted how brain cancer is treated. Researchers have devised new ways to administer medications locally, improving therapy effectiveness and patient outcomes. Hydrogels are flexible bases that can hold different therapeutic agents, such as chemotherapeutics and nanoparticles. They can be released slowly and work specifically on tumor sites.

Chen et al. worked on a light-activated hydrogel system that targets tumors using mesoporous silica nanoparticles (MSNs). This theranostic platform showed that it could deliver drugs to tumor tissues sustainably and effectively, making it a real-life anticancer treatment [207]. The study’s results showed that the hydrogel could effectively release doxorubicin, which caused a significant decrease in tumor size in preclinical models. This proved that the hydrogel could be a therapeutic agent for treating brain tumors.

Sustained medication release is crucial for effective tumor therapy, and hydrogel systems can deliver it. One example is the work of Han et al., who showed that chitosan hydrogels loaded with gemcitabine might reduce tumor recurrence and toxicity with a longitudinal anticancer impact achieved through sustained drug release [208]. In a similar vein, Wang et al. improved the therapeutic result in tumor models by creating a thermoresponsive injectable hydrogel that mixed doxorubicin with polydopamine nanoparticles, demonstrating efficient drug retention and controlled release [209]. These results highlight the possibility of hydrogels as a means to enhance the efficacy of medication delivery in the treatment of brain tumors.

Hydrogel matrices have already demonstrated improved therapeutic efficacy when combined with nanoparticles. For instance, Kiseleva et al. [210] combined gold-enhanced brachytherapy using a hydrogel that releases nanoparticles to improve imaging and treatment precision. Hydrogels containing mesoporous silica nanoparticles have synergistic benefits, as pointed out by Zhou et al. which allow for tailored drug delivery and circumvent the drawbacks of conventional systemic treatments [211]. Nanoparticle integration allows for multifunctional treatment techniques such as immunotherapy and photothermal therapy and improves drug loading capacity.

In addition, hydrogels overcome obstacles presented by the tumor microenvironment, which frequently blocks the effective penetration of drugs. According to Kong et al., smart hydrogel nanoparticles can reduce the extracellular matrix’s size, enhancing drug delivery into tumor tissues [212]. In addition, Shen et al. [213] demonstrated a new way to improve treatment results by investigating the effectiveness of hydrogel systems loaded with losartan to decrease tumor collagen levels and increase nanoparticle penetration. This demonstrates how versatile hydrogel systems are for changing the tumor microenvironment to improve medication delivery.

Hydrogels have the potential to transport drugs and react to environmental cues such as pH and temperature, which are common in tumors. One example is Yang’s talk about intelligent hydrogels, which have the potential to deliver targeted therapy using controlled drug release mechanisms and avoid the BBB [184]. Likewise, new pH/temperature dual-sensitive hydrogels have the potential to improve the efficiency of chemo-photothermal combinations by enabling the targeted destruction of tumor cells with reduced systemic toxicity [214].

Finally, improvements in hydrogel-based nanoparticles are a big step forward in the fight against brain cancer. These methods improve targeted drug delivery and therapeutic effectiveness and offer new ways to deal with the problems that the tumor microenvironment causes. As the study moves forward, combining hydrogels with cutting-edge nanotechnology could significantly improve the outcomes of brain tumor treatments for patients.

### Combination therapies

**Gene therapy:** A noteworthy advancement involves the exploration of hydrogel-based nanoparticles in gene therapy applications. These nanoparticles exhibit the capability to safeguard genetic material during delivery and release it in a controlled manner at the designated target site. This innovative approach holds potential for treating genetic disorders and cancers by facilitating the direct delivery of therapeutic genes to cells, paving the way for more precise and targeted therapeutic interventions [215].

**Immunotherapy enhancement:** In the landscape of cancer treatment, hydrogel-based nanoparticles are playing a pivotal role in augmenting the efficacy of immunotherapies. These nanoparticles can be laden with immunostimulatory agents and cytokines, empowering the immune system to recognize and eradicate cancer cells more effectively. This application underscores the potential of hydrogel-based nanoparticles as versatile tools in advancing immunotherapeutic strategies for cancer treatment [216].

### Potential developments and new applications in hydrogel-based nanoparticles

**Expanding horizons in gene therapy:** Hydrogel-based nanoparticles are emerging as ideal carriers for gene therapy applications. Leveraging their biocompatibility and adeptness in encapsulating nucleic acids, these nanoparticles can be tailored to target specific cells and precisely release their payload in a controlled manner. This advancement positions hydrogel-based nanoparticles as promising and versatile tools for the treatment of a diverse array of diseases through the avenue of gene therapy [217].

The capacity of hydrogel-based nanoparticles to protect nucleic acids from degradation while facilitating their delivery to target cells represents a notable benefit. For instance, Ma et al. developed hydrogel nanoparticles that react to reductive environments, demonstrating efficient systemic delivery of small interfering RNA (siRNA) in vivo, leading to significant luciferase gene silencing in HeLa cells. Ma et al. (2015). This study highlights the ability of hydrogels to serve as effective carriers for RNA-based therapies, ensuring that the therapeutic agents remain intact until they reach their target location. Moreover, the ability to tailor the properties of hydrogel nanoparticles enhances their efficacy in gene therapy. Conde and colleagues created an implantable hydrogel that incorporates gold nanoswitches, enabling the detection and management of cancer multidrug resistance (MDR). This innovative approach addresses the challenge of drug resistance while also showcasing the potential of hydrogels to be integrated with other nanomaterials to enhance therapeutic efficacy [185, 218].

**Harnessing immunotherapy with hydrogel-based nanoparticles:** Hydrogel-based nanoparticles are proving instrumental in the realm of immunotherapy. These nanoparticles can serve as carriers for immunotherapeutic agents, releasing them strategically within the tumor microenvironment. This targeted release mechanism enhances the body’s immune response against cancer cells, showcasing the potential of hydrogel-based nanoparticles in fortifying immunotherapy strategies against cancer [219].

One of the main benefits of hydrogel-based nanoparticles is their ability to provide sustained and localized release of immunotherapeutic agents in the tumor microenvironment. Liu et al. indicate that the properties of hydrogels, such as their injectability, degradability, and stimuli-responsive characteristics, facilitate the effective encapsulation of a range of immunotherapeutic agents, including cytokines and antibodies, which can elicit strong immune responses against tumors. The localized delivery plays a vital role in improving the therapeutic index of immunotherapy, simultaneously minimizing off-target effects that may arise from systemic administration. Furthermore, the integration of immunoadjuvants into hydrogel systems has the potential to greatly enhance the immune response. For example, Chen et al. showed that the integration of photothermal therapy with immune-adjuvant nanoparticles resulted in elevated serum levels of Th1 cytokines, including TNF-*α* and IFN-*γ*, which are essential for successful cancer immunotherapy. This collaborative strategy not only improves the immune response but also encourages tumor regression, highlighting the promise of hydrogel-based nanoparticles in combination therapies [220, 221].

The combination of hydrogel-based nanoparticles with additional therapeutic approaches, including photothermal therapy and checkpoint blockade, significantly increases their efficacy in cancer treatment. Liu et al. emphasized the efficacy of integrating injectable supramolecular hydrogels with immune checkpoint inhibitors, showcasing enhanced results in cancer chemo-immunotherapy. This combined approach utilizes the advantages of various therapies to attain a more effective anti-tumor response [222].

#### Innovative clinical trial designs for hydrogel-based nanoparticles in brain tumor therapy

Bayesian Optimal Interval (BOIN) Design: The Bayesian optimal interval (BOIN) design represents a cutting-edge approach in the development and evaluation of hydrogel-based nanoparticles for brain tumor therapy. This design stands out for its flexibility and adaptability, making it applicable to various types of early phase trials. Whether it involves dose-finding, dose-expansion, or combination trials, the BOIN design provides a robust framework for conducting clinical trials with hydrogel-based nanoparticles, contributing to the advancement of brain tumor therapy [223]. The BOIN design is based on a simple algorithm that assigns patients to the most promising dose level, based on the observed toxicity and efficacy outcomes. The BOIN design has been shown to outperform the conventional 3 + 3 design in terms of ethical and statistical properties, such as minimizing the number of patients treated at suboptimal or toxic doses, maximizing the probability of selecting the optimal dose, and reducing the trial duration and sample size [224]. Another novel approach is the phase 0 trial design, which is a first-in-human study that involves the administration of microdoses of a new drug to a small cohort of patients. The objectives of phase 0 trials are to assess the pharmacokinetics (PK) and pharmacodynamics (PD) of the drug in the human body, by measuring the drug concentration in the blood and the tumor tissue, and the drug effect on the molecular and cellular targets. Phase 0 trials can provide valuable information on the feasibility and safety of a new drug, and facilitate the decision-making process for subsequent phase I trials [225]. The clinical application of hydrogel-based nanoparticles for brain tumor treatment involves not only scientific and technical aspects but also regulatory and ethical considerations. Clinical trials are strictly regulated to protect participants and ensure reliable results. Key to these trials are eligibility criteria, which are based on factors, such as diagnosis, tumor stage and grade, previous treatments, performance status, and patient comorbidities. These criteria aim to balance scientific rigor, study generalizability, and patient benefits and risks. Adherence to neuro-oncology practice standards and guidelines, such as those provided by the Congress of Neurological Surgeons, and the American Association of Neurological Surgeons (AANS), is another crucial aspect of these trials. These guidelines offer best practices for diagnosing, treating, and following up with patients with CNS tumors [41].

## Conclusion

Recent progress in the development of hydrogel-based nanoparticles for treating brain tumors represents a significant leap forward in the field of nanomedicine. These innovative drug delivery systems hold the potential to revolutionize the current approach to brain tumor management, addressing challenges related to therapy specificity, delivery efficiency, and patient morbidity. Customizable hydrogel nanoparticles have emerged as versatile agents capable of overcoming the formidable blood–brain barrier (BBB). They enable targeted delivery of therapeutic compounds and serve as platforms for tissue regeneration.

Research has demonstrated that hydrogel nanoparticles not only facilitate localized drug delivery to tumor cells but also contribute to breakthroughs in areas, such as immunotherapy and gene therapy. Their responsiveness to biological signals ensures the targeted release of drugs, minimizing systemic toxicity and enhancing treatment efficacy. Furthermore, the potential of combination therapies offers personalized and multimodal treatment strategies.

Despite these remarkable advancements, technical and biological challenges remain. These include the synthesis of stable and reproducible nanoparticles, scalability, and fine-tuning targeting mechanisms to overcome the high interstitial pressure within brain tumors. Biocompatibility and regulatory approvals continue to be primary concerns. Future research should focus on optimizing hydrogel-based nanoparticles for clinical use by refining targeting capabilities, exploring stimuli-responsive and biodegradable materials, and utilizing advanced manufacturing techniques.

Innovative clinical trial designs, such as the brain tumor consortium (BOIN) and expanded computational models, will accelerate the translation of these innovations to patients. The integration of technologies such as 3D bioprinting and the exploration of novel biocompatible materials suggest that interdisciplinary approaches will accelerate the development of effective brain tumor therapies. Researchers must remain committed to enhancing our fundamental understanding of these materials, the interaction between nanoparticles and biological systems, and the clinical implications of their use.

In conclusion, the strategic utilization of hydrogel-based nanoparticles holds a promising future for brain tumor management. With continued advancements and collaborative efforts in research, development, and clinical trials, there is an optimistic vision for successfully integrating these systems into standard care practices, ultimately leading to improved outcomes for brain tumor patients worldwide.

## Data Availability

No datasets were generated or analysed during the current study.
